# Calcite Tailoring by Limpets: The Prismatic and Acicular-Foliated Microstructures of Members of The Nacellidae

**DOI:** 10.1021/acs.cgd.5c01236

**Published:** 2026-01-15

**Authors:** Katarzyna Berent, Marta Gajewska, Antonio G. Checa

**Affiliations:** † Academic Centre for Materials and Nanotechnology, 49811 AGH University of Krakow, 30 Mickiewicza Avenue, Krakow 30-059, Poland; ‡ Departamento de Estratigrafía y Paleontología, 16741 Universidad de Granada, Fuentenueva Street n/n, Granada 18071, Spain

## Abstract

Limpets of the family Nacellidae construct their shells with an outer prismatic layer, underlain by an acicular-foliated layer, and occasionally an internal crossed-lamellar layer. The outer layer consists of prisms oriented approximately perpendicular to the shell growth surface. At the transition to the acicular-foliated layer, these prisms subdivide into segments that become incorporated into the underlying folia. The folia are extensive, radially oriented, and further subdivided into transverse acicles, hence the term “acicular-foliated”. Crystallographically, the outer prisms are remarkable in having their *c*-axes oriented at a high angle to their elongation and aligned toward the shell margin, with one *a*-axis typically perpendicular to the growth surface. The prisms exhibit a high degree of co-orientation, with textures ranging from diffuse to well-organized sheet textures. When considering rhombohedral face orientations, the texture may appear either single- or double-crystal-like. This crystallographic arrangement is inherited by the acicular-foliated layer, where the texture invariably becomes stronger. Such an unusual orientation likely results from oriented nucleation on an organic template, possibly the mantle surface, combined with crystal competition in the direction toward the shell margin. From both morphological and crystallographic perspectives, the prismatic-acicular-foliated microstructural complex of nacellids is unparalleled among molluscs.

## Graphical abstract

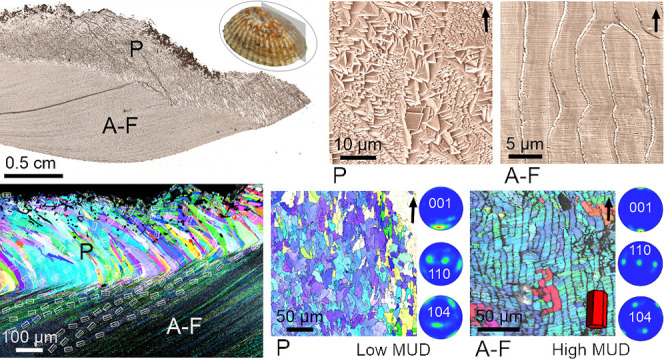

## 1 Introduction

The first general account of molluscan shell microstructures was provided by Bøggild.^1^ Despite relying on optical microscopy, his work revealed the wide array of microstructures produced by molluscs. This repertoire expanded further with the advent of electron microscopy techniques,^2–5^ reaching at least twelve basic microstructural types composed of either calcite or aragonite.^6^ Based on current data, it is safe to assert that molluscs are the most skilled producers of calcium carbonate-based mineralized tissues in the animal kingdom. Some microstructures are relatively simple (e.g., fibrous aragonite, granular calcite), while others display high degrees of sophistication (e.g., nacre, columnar prismatic, crossed-lamellar, and fibrous helical). We are now beginning to understand the production strategies behind them, which involve biomolecules, self-organization, and mantle cell activity.^6,7^ Moreover, their biocomposite structure endows these materials with superior mechanical properties, including increased strength and toughness: the stiff mineral phase provides hardness and load-bearing capacity, while the ductile organic layer enhances energy absorption, crack deflection, and fracture resistance.^8,9^

However, the range of molluscan microstructures becomes even broader when fossil data are considered, indicating that certain microstructures evolved independently across molluscan classes. The most striking examples are nacre and crossed-lamellar microstructures, the two most widespread types. Both were absent during the Cambrian, when all conchiferan classes, except scaphopods, emerged. Nacre subsequently appeared in cephalopods, bivalves, and gastropods during the Late Ordovician,^10^ and it is also present in modern monoplacophorans.^5^ These findings suggest that nacre evolved independently in each class, as evidenced by differences in tablet arrangement and growth patterns of lamellae. A similar case applies to crossed-lamellar microstructures, which likely developed independently in gastropods and bivalves. Additionally, scaphopods may have inherited their crossed-lamellar microstructure from their conocardioid rostroconch ancestors, which possessed internal crossed-lamellar layers.^11^ A third example is the foliated calcite microstructure, described in Cambrian helcionelloids and rostroconchs,^12,13^ but absent in the earliest bivalves and gastropods. Foliated calcite is now widespread and has been extensively studied in pteriomorph bivalves (particularly within the order Pectinida and the superfamily Ostreoidea, order Ostreida). It is also present in limpet gastropods of the family Nacellidae, and in a couple of additional instances of limpets.^14–16^ Accordingly, this microstructure appears to have evolved independently at least four times within the Mollusca.

Limpets (gastropod subclass Patellogastropoda) are remarkable for the wide variety of microstructures they can produce, especially given their limited representation within Gastropoda (360 versus 40,430 currently accepted marine gastropod species).^17^ Their shells typically consist of four to six microstructurally distinct layers.^14^ In most cases, these shells feature external calcitic layers and internal aragonitic layers, although exceptions exist. The few available studies reveal an impressive diversity of microstructures.^14,16^ The external calcitic layers may include various prismatic, foliated, and crossed-foliated types, the latter two being unique to this group within Gastropoda. The internal aragonitic layers generally consist of crossed-lamellar or complex crossed-lamellar microstructures, crossed by the prismatic myostracum.^14^ In his comprehensive review, MacClintock^14^ identified seventeen patellogastropod groups based on different combinations of superimposed layered microstructures.

Among gastropods, only the patellogastropod family Nacellidae (superfamily Patelloidea),^14^ the Acmaeidae *Acmaea mitra*,^15^ and the Neolepetopsidae *Eulepetopsis vitrea* ^16^ (Superfamily Lottioidea) possess a foliated microstructure. In Nacellidae, this microstructure was described as consisting of imbricated folia arranged subparallel to the inner shell surface and elongated along the local radial direction.^14^ In this family, there is always an external layer of prisms that elongate perpendicular to the growth surface, grading inward into the foliated layer. We analyzed the foliated and associated prismatic layers in species from the two main genera of Nacellidae, *Cellana* and *Nacella*, which include 39 and 13 accepted living species, respectively (the third genus, *Naccula*, with only three species, was not included).^17^ Scanning electron microscopy (SEM) revealed notable differences between the nacellid foliated microstructure and that of other molluscs, particularly bivalves. Due to these peculiarities, we propose a new term for this structure: acicular-foliated (A-F). Based on electron backscatter diffraction (EBSD) analyses, we conclude that both the prismatic and A-F layers share similar crystallographic organization and texture, clearly distinct from those of comparable calcitic materials in or outside molluscs. Notably, the organization of the A-F microstructure is inherited from that of the prismatic microstructure, indicating a biologically regulated transition between structurally distinct layers. This study highlights the remarkable adaptability of limpets in engineering their mineralized structures. It also increases the variety of fabricational strategies of known biogenic calcitic structures, further emphasizing the contrast between biological and geological mineralization processes.

## 2 Material and Methods

### 2.1 Material

We used shells of the following species of Nacellidae: *Cellana toreuma* (2 specimens, Hokkaido, Japan), *C. tramoserica* (1 specimen, Wool Bay, South Australia), *C. testudinaria* (2 specimens, Negros Island, Philippines), C. sp. (possibly *C. toreuma* or *C. testudinaria*, 3 specimens, Japan, locality unknown), *Nacella delesserta* (1 specimen, South Africa, locality unknown), *N. concinna* (19 specimens, Isla Rey Jorge, Antarctica; 9 specimens, Yelcho, South Chile), and *N. deaurata* (4 specimens, San Isidro, Strait of Magellan). A fragmentary specimen of *E. vitrea* (East Pacific Rise) (Neolepetopsidae, Lottioidea) was also analyzed.

### 2.2 SEM

Fragments of the internal shell surfaces were mounted, carbon-coated using an Emitech K975X carbon evaporator, and observed with field emission gun scanning electron microscopes (FEG-SEMs): a Zeiss Auriga and an FEI QemScan 650 F at the Center for Scientific Instrumentation (CIC), University of Granada (UGR), Spain, and an FEI Versa 3D at the Academic Centre for Materials and Nanotechnology (ACMiN), AGH University of Krakow, Poland.

### 2.3 TEM

The focused ion beam (FIB) lift-out method was used to extract a cross-sectional lamella of the A-F layer of *C. toreuma*, using an FEI Quanta 3D 200i dual beam SEM. The lamella was cleaned by an ion beam at 2 kV to remove the surface damage, namely amorphization and Ga^+^ ions implantation layer. Bright-field (BF) imaging and selected area electron diffraction (SAED) analyses were conducted on the lamella using an FEI Tecnai TF 20 X-TWIN transmission electron microscope (TEM) operated at 200 kV. All the above equipment is housed in the ACMiN.

### 2.4 EBSD

EBSD analyses were performed to identify the crystallographic structure of the shell layers. Fragments from all *Cellana* species and *Nacella concinna* were embedded in epoxy resin and sectioned perpendicular to the shell surface. Samples were then ground and polished using a standard metallographic preparation route: mechanical polishing with silicon carbide papers of increasing grit sizes (320, 500, 800, 1200, 2000, and 4000), followed by polishing with a diamond suspension of 3 μm, 1 μm, and 0.25 μm. Final polishing was performed for 1 min with colloidal silica using a Struers Tegramin-25 automatic polisher. EBSD maps from the internal shell surfaces, both polished and unpolished, were also obtained. The FIB prepared lamella used for TEM was also analyzed by transmission EBSD (*t*-EBSD). These maps provided useful information about the shapes and crystallographic orientations of calcite laths. However, the quality of the maps on raw growth surfaces was consistently lower, due to the EBSD technique’s sensitivity to surface topography (roughness). EBSD measurements were carried out with an FEI Versa 3D FESEM, equipped with an EDAX Hikari XP and an Oxford Instruments Symmetry S2 camera. EBSD maps were collected at an accelerating voltage of 12–15 kV under low vacuum mode (40 Pa of H_2_O). Data were analyzed with OIM Data Collection V. 7.3 and Aztec 6.1 software. The EBSD data are presented as Pole Figure (PF), Inverse Pole Figure (IPF) maps, and Kernel Average Misorientation (KAM) maps. PFs show the distribution of specific crystallographic planes, revealing texture patterns related to preferred crystal orientations during growth. IPF maps illustrate how grain orientations vary relative to the growth direction, providing insight into changes in crystal alignment layer by layer. KAM maps highlight local misorientations within grains, indicating zones of strain and the presence of low-angle boundaries. The multiples of uniform distribution (MUD) value was used to quantify the strength of the material’s crystallographic texture, that is, the degree of crystal co-orientation. MUD values express the ratio between the observed density of orientations in an EBSD pole figure and the density expected for a material with a fully random, uniform orientation distribution. A value of 1 indicates random orientation, whereas higher values denote progressively stronger textures. Values ≥ 700 indicate nearly perfect crystal co-orientation. Two grain-size descriptors were applied to characterize the microstructures of the shell layers. The equivalent circle diameter (ECD) provides an area-normalized grain-size measure that is independent of shape, allowing direct comparison across crystalline units with differing morphologies. In contrast, the maximum Feret diameter captures the longest dimension of individual grains and is especially informative for describing highly elongated or acicular morphologies. Disorientation angle distributions (DADs) were also calculated to assess crystallographic orientation relationships between neighboring pixels or reconstructed grains, and to quantify the relative abundances of low-angle, high-angle, and twin-related boundaries.

### 2.5 Nanoindentation Analysis

Nanoindentation tests were performed both parallel and perpendicular to the shell surface. In the first approach, surface nanoindentation was carried out on the internal surfaces of both the internal and external shell layers using a Hysitron TI Premier system with a diamond Berkovich three-sided pyramid indenter operated in load-controlled mode at a maximum load of 2 mN. The loading protocol consisted of a 15 s loading segment, a 10 s dwell at maximum load, and a 15 s unloading segment. A hydrothermal calcite crystal polished along {100} was used as a reference standard. Samples from the prismatic and A-F layers of *N. concinna* were analyzed under these conditions, both in dry conditions and after immersion in Milli-Q water for 20 h. These surface measurements were performed at the CIC (UGR). In the second approach, nanoindentation was performed on polished cross sections using a diamond cube-corner indenter operating in continuous stiffness measurement (CSM) mode. These tests were performed in the FEI Versa 3D FESEM using a FemtoTools FT-NMT04 nanoindenter in displacement control, up to a maximum indentation depth of 500 nm, at a loading rate of 50 nm/s. The CSM configuration enabled acquisition of hardness and reduced modulus profiles as a function of depth, and average mechanical values were calculated over the interval of 200–450 nm, where the response was stable and free from surface artifacts. Under these configurations, a hydrothermal calcite crystal sample and both the prismatic and A-F layers of *C. toreuma* were examined. This second set of measurements was conducted at the ACMiN.

## 3 Results

### 3.1 Shell Microstructures

#### 3.1.1 Prismatic Layer

In all examined species of *Cellana* and *Nacella*, the sequence of calcitic shell layers is very similar. The main difference between the two genera lies in the presence of internal crossed-lamellar layers in *Cellana* (Figure 1). In cross-section, the shell has an outer calcitic prismatic layer composed of long prisms that elongate perpendicular to the growth lines (Figure 2a–e). This microstructure has been described either as “complex-prismatic”^14^ or “simple prismatic type-A”.^15^ This outer layer transitions abruptly into a seemingly foliated layer (A-F layer, see below; previously described as “foliated”^14^ or “regularly foliated”^15^), without any neat interruption (Figure 2c). Both layers increase in thickness toward the shell margin, but the A-F layer is consistently thicker (about twice as thick in *C. toreuma*, Figure 2a,b, and up to three times thicker in *C. tramoserica*, Figure 2d) compared to the outer prismatic layer. The outer prismatic layer is often bioeroded, particularly toward the apex (Figure 2a, b), where it may even disappear. In *Nacella*, the prismatic and A-F layers constitute the entire shell structure, and the myostracum passes though the A-F layer. In contrast, *Cellana* exhibits two additional internal crossed-lamellar layers,^14^ which taper out toward the shell margin and are intersected by the myostracum (Figure 2b,d).

**Figure 1:**
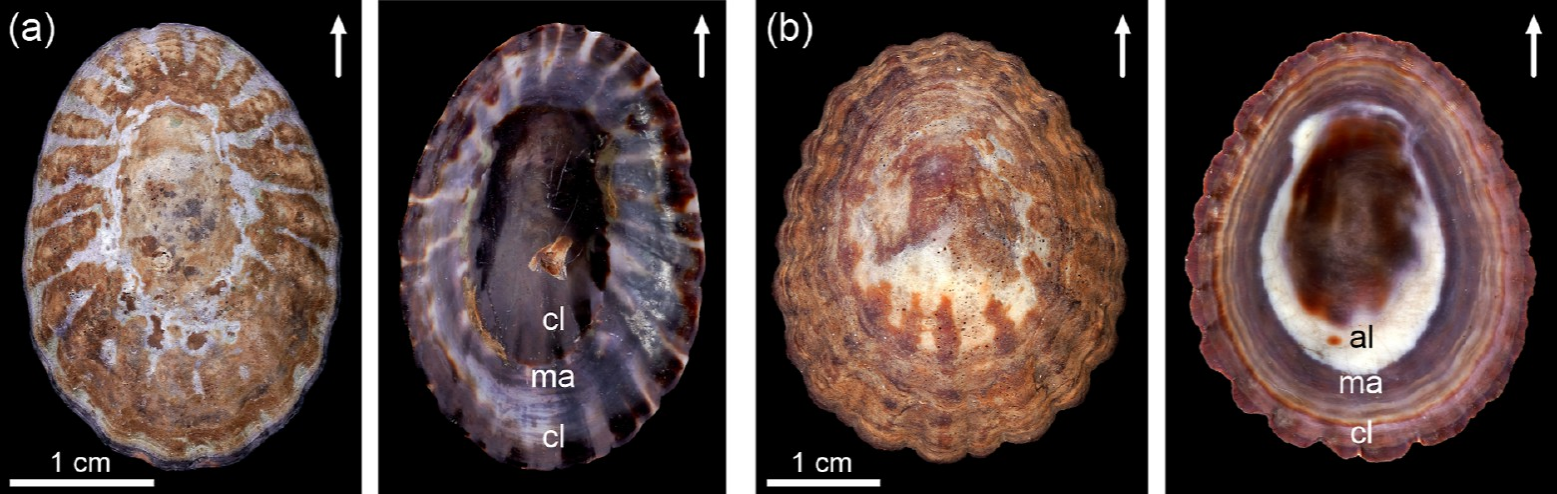
External and internal views of specimens of (a) *Nacella concinna*, and (b) *Cellana* sp. Arrows indicate the anterior direction. al: aragonitic (crossed-lamellar) layers; cl: calcitic layers, ma: muscle attachment.

**Figure 2:**
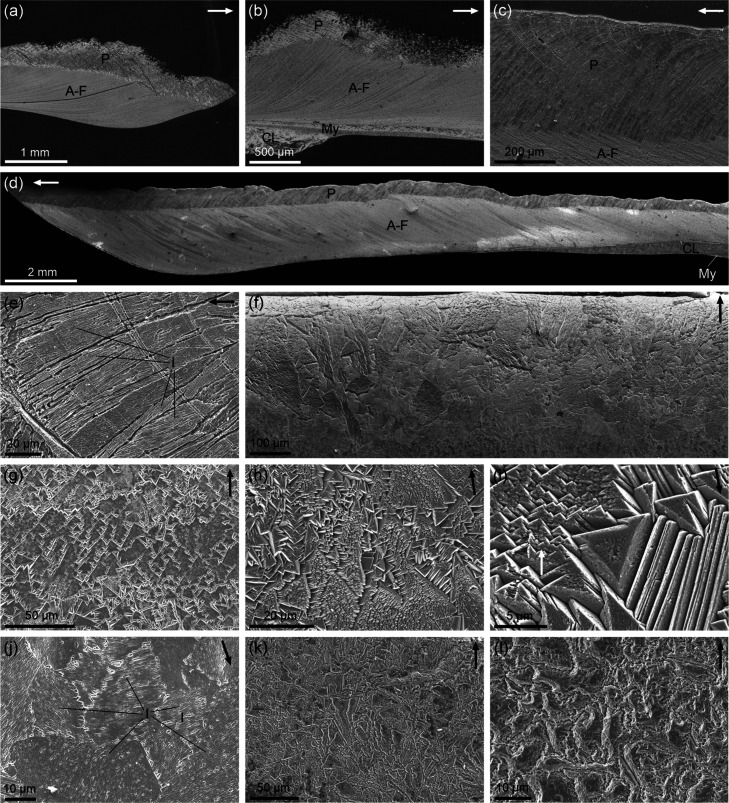
General shell structure (a–d) and prismatic layer (e–l). (a) *Cellana toreuma*. Longitudinal section of the marginal area. (b) *Cellana toreuma*. Longitudinal section of the central shell area showing all layers. (c) *Cellana testudinaria*. View of the prismatic layer and the transition to the A-F layer. Prisms curve to remain approximately perpendicular to growth lines. (d) *Cellana tramoserica*. Longitudinal section of the marginal and middle shell areas, with indication of shell layers. (e) *Cellana testudinaria*. Detail of the prisms. Note internal longitudinal lineations. (f) *Nacella concinna*. General view of the internal surface of the margin, composed of large grains elongated in the direction of the margin (arrow). (g) *Cellana toreuma*. The prismatic layer is made of rhombohedral units with high coalignment. (h) *Cellana toreuma*. Note the general elongation of prisms with rhombohedral outlines in the direction of the margin. (i) *Cellana toreuma*. Detail of rhombohedral units. (j) *Nacella concinna*. View of the growth surface of the prismatic layer. Note internal lineations of grains. In (g), (h), and (i), grain edges extend toward the shell interior along steep rhombohedral surfaces, providing partial 3D views of the complex fractal-like rhombohedral morphologies. Conversely, in (j), grains meet directly at the growth surface. (k) *Cellana* sp. Grains have irregular outlines but jagged edges. (l) *Cellana testudinaria*. Highly irregular outlines, with slightly dendritic edges. A-F: A-F layer, CL: crossed-lamellar layer, l: lineations, my: myostracum, P: prismatic layer. Arrows indicate the direction toward the margin. (a) to (e) are longitudinal sections; (f) to (l) are views of growth surfaces.

The prisms of the outer layer are invariably oriented at a high angle to the growth margin. When the margin curves back toward the outer shell surface, the prisms adopt a fan-like arrangement to accommodate to the changing orientation of the growth surface (e.g., *C. testudinaria*, Figure 2c). In polished-etched cross sections, the prisms display internal lineations parallel to their elongation axis (Figure 2e). Prisms are uneven in size and may intersect to some extent.

On the growth surface, prisms appear highly irregular in outline and size across all species (Figure 2f–l). They may exhibit angular, faceted shapes with heavily jagged boundaries (Figure 2f–j), or irregular, slightly dendritic boundaries (Figure 2k,l). Prism sizes may change from several hundreds (Figure 2f) down to a few microns (Figure 2l). Even within a single shell, prism size and shape vary significantly across regions (e.g., Figure 2f,k,l). Prisms display internal parallel lineations whose orientation changes from prism to prism (Figure 2j), indicating their crystallographic nature. In some species (e.g., *C. toreuma*, *N. concinna*, Figure 2f–i), triangular features are abundant. These often have edges that correspond to distinct crystal faces oriented at high angles to the growth surface (Figure 2h,i), suggesting that they are rhombohedra truncated by the shell surface (Figure 2g–i). In some cases, these triangular features exhibit a high degree of co-orientation (Figure 2g). Prism interiors may be studded with smaller, lower-order triangular features, producing a fractal-like rhombohedral landscape (Figure 2g–i). In other instances, the grain edges meet directly on the growth surface (Figure 2j). In some cases (Figure 2f,h), prism outlines appear neatly elongated in parallel to the direction of the margin.

#### 3.1.2 Transition Layer

Toward the shell interior, the prismatic layer gradually changes into the A-F layer through a transitional layer. In longitudinal section, the transition surface is irregular and stepped, and the prisms progressively subdivide into thinner units (Figure 3a). On the growth surface, toward the shell interior, the prisms of the outer layer become covered by calcite tablets, which are initially irregular and later assume a rhomboidal shape, sometimes growing in a spiral pattern (Figure 3b,c). In the specimen of *N. concinna* of Figures 2f, and 3d–g, the large prisms at the outer margin gradually transform into smaller units toward the shell interior (Figure 3e), which subsequently develop into radial rows of fused small rhombohedra (Figure 3f). Eventually, folia begin to appear, coexisting with residual rhombohedral rows (Figure 3g), which progressively fade toward the interior (bottom of Figure 3d).

**Figure 3:**
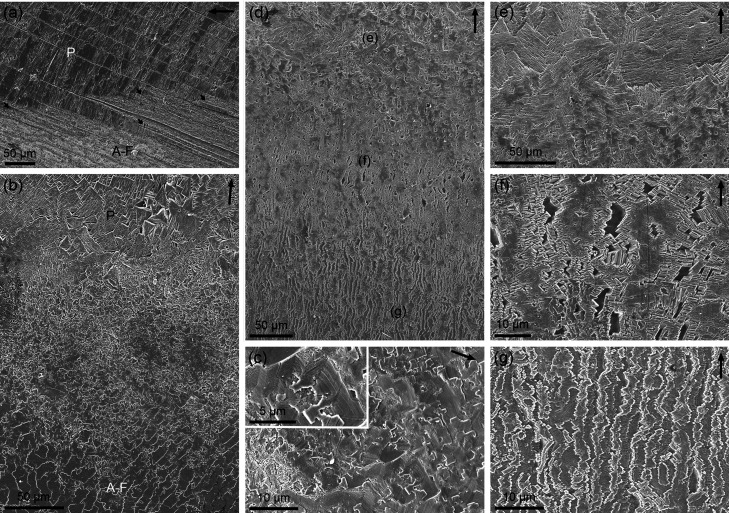
Transition between the outer prismatic and A-F layers. (a) *Cellana testudinaria*. Boundary between the prismatic and A-F layers. Prisms become partitioned into progressively thinner layers (short arrows). (b) *Cellana toreuma*. Transition from the prismatic to the A-F layer, with an intermediate layer of tabular calcite. (c) *Nacella deaurata*. Detail of tabular calcite at the transition. (d–g) *Nacella cocinna*, same specimen as in Figure 2f. (d) General view of the transition. (e–g) Details of the areas indicated in (d). (e) Initial partitions of prisms, (f) advanced stage, (g) initiation of the A-F layer, with the edges of folia delineated by rhombohedra like those seen in (f). A-F: A-F layer, P: prismatic layer. Arrows in the top right corner indicate the direction toward the shell margin.

#### 3.1.3 A-F Layer

At the transitional zone, the prismatic segments organize into continuous imbricated laminae or folia that become radial (i.e., perpendicular to the margin) (Figures 3b,d, and 4a), sometimes after a brief initial oblique phase. The folia dip toward the shell interior at a very shallow angle and imbricate with each other (Figure 4a–f). A quick examination of the shell interior of 18 specimens of *N. concinna* reveals no consistent imbrication direction. The margins of the folia may grow toward either the anterior (∼33%) or the posterior (∼41%) ends of the animal. In some cases (∼16%), the growth direction changes over short distances, while in others (∼10%), the folia orient parallel to the margin. Folia typically form regular arrangements, with their lateral (growth) margins being linear or wavy, the latter usually due to the introduction of new folia (Figure 4a–e). Their widths exposed on the growth surface are typically in the range of 10–15 μm. Occasionally, areas with irregular or highly irregular folia distribution and variable widths are observed (e.g., Figure 4f). The lengths of individual folia in the radial direction vary, usually spanning several hundred microns (Figure 4a). Sometimes, they have wide rhomboidal (Figure 4c), rarely spiral (Figure 4b) endings, oriented both internally and externally. On intact surfaces (Figure 4d–f, thick short arrows), as well as in delaminated areas of the A-F layer (Figure 4g, arrows), initiation points of new folia forming through stepped growth from underlying folia can be observed. Immediately after a folium terminates or a new one initiates, the two adjacent folia shift closer together or farther apart, respectively, adjusting the spacing so that the step size in the circumferential direction remains approximately constant (Figure 4b–d, f). Profiles of folia detached from the shell show widths of about 0.3 μm (Figure 4h).

**Figure 4:**
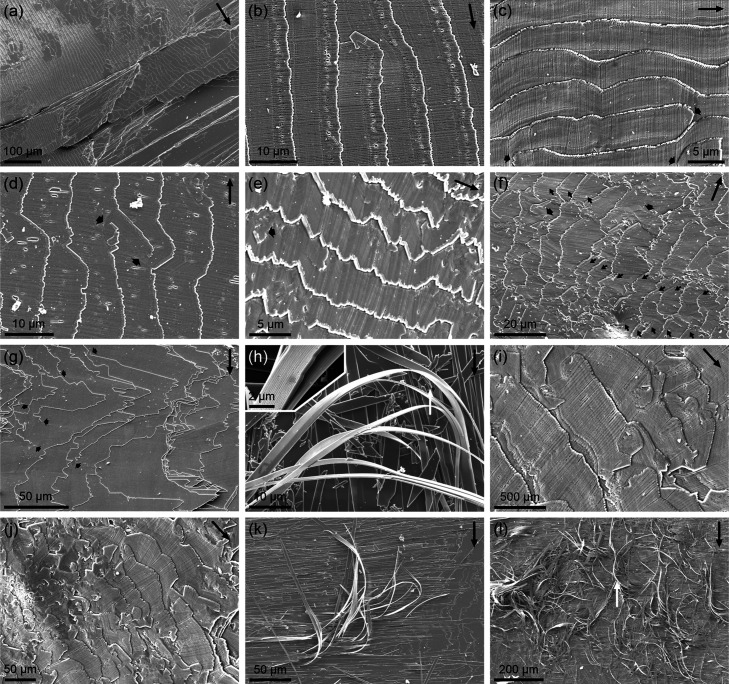
A-F layer. (a) *Cellana toreuma*. Radial arrangement of folia. (b) *Cellana toreuma*. Regular arrangement of folia and their constituting acicles. The central folium shows a spiral ending. (c) *Nacella deaurata*. Modification of folia arrangement to accommodate new folia. Thick arrows indicate rhomboidal side endings. (d) *Cellana toreuma*. Wavy arrangement of folia due to the emergence of new folia from underlying ones. (e) *Nacella concinna*. Folia conisting of wide acicles with arrowhead endings. The wide arrow points to an incipient folium. (f) *Cellana toreuma*. Irregular arrangement of folia. Thick arrows point to folia originating from underlying ones. Sequences of thin arrows point to similarly sized/oriented acicles. (g) *Nacella concinna*. Mechanically exfoliated shell. Note folia extensions toward the shell interior and stepwise folia initiation (arrows). (h) *Nacella concinna*. Groups of mechanically exfoliated acicles. Their bending denotes high flexibility. (i), (j) *Nacella deaurata*. Irregular folia. Sets of acicles with totally different shapes and orientations. (k), (l) *Nacella concinna*. Exfoliated, split acicles forming dense bundles (l). Arrows in the top right corner indicate the direction to the margin.

The most characteristic feature of folia is that they are composed of juxtaposed narrow, lath-like elements of variable widths (0.2 to >1.5 μm), which extend at a high angle to the margins of the folia and terminate in slightly acute arrowhead-shaped ends (Figure 4b–f,i,j), giving the folia margins a serrated appearance. We refer to these elements as acicles, owing to their extreme thinness. Due to the presence of these transverse acicles, unprecedented in other foliated materials, we define this microstructure as acicular-foliated (A-F).

Folia display distinct growth lines parallel to their margins, visible in superposed folia and continuing across adjoining laths (Figure 4b–f,i,j). In regularly arranged folia, the acicles are aligned in parallel within a single folium (Figure 4b–d). In irregularly distributed folia, the acicles tend to occur in clusters of parallel elements with varying orientations relative to adjacent groups, leading to mutual interference (Figure 4e,f). These clusters tend to replicate, though not strictly, across superposed folia (Figure 4f). Occasionally, tablets of varying sizes and unusual orientations appear among the acicles (Figure 4e,i,j). They have straight margins and may sometimes be spiral in shape.

In areas where the folia have mechanically detached, their exposed surfaces on the shell interior may extend for hundreds of microns (Figure 4g). When individual acicles split, they extend over comparable lengths (Figure 4h,k). In some cases, they form a dense tangle, resembling bundles of threads curling in all directions (Figure 4l), highlighting their extreme flexibility. Notably, these features were observed in freshly captured, wet animals.

### 3.2 TEM Observation of the A-F Microstructure

TEM imaging of the FIB-prepared *C. toreuma* lamella was performed using the high-angle annular dark-field scanning transmission electron microscopy (HAADF-STEM) mode. The sample was sectioned perpendicular to the shell growth surface, allowing estimation of folia thickness, which range from approximately 0.1 to 0.5 μm (Figure 5a). The interfaces between superposed folia appear as narrow, bright gaps a few nanometers wide, which may correspond to amorphous (possibly organic) interphases. In contrast, acicles within individual folia are tightly joined, with no visible interacicle gaps (short arrows in Figure 5b,c). These acicles possess broadly open arrowhead-shaped ends that are similarly shaped and aligned within each folium. The spacing between them may vary depending on their width or orientation relative to the sectioning plane. Longer arrows in Figure 5b indicate the side edges of particular folia. Crystallographic indexing in Figure 5a shows that the *c*-axis lies in the plane of the analyzed acicles and is shared among acicles with a similar diffraction contrast.

**Figure 5:**
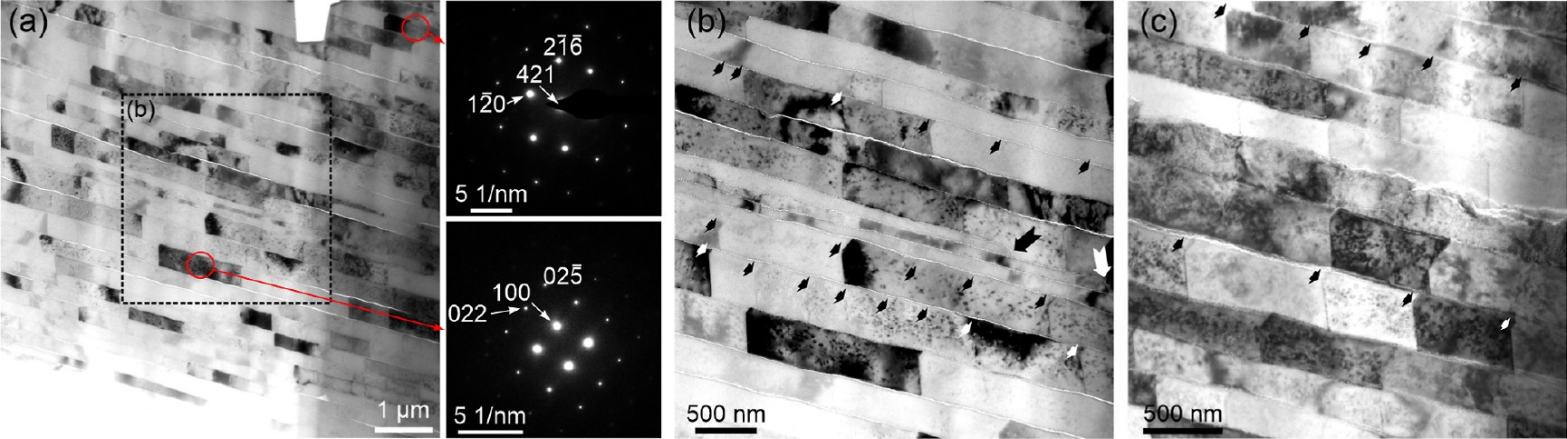
TEM examination of an FIB-prepared cross-section of the A-F layer of *Cellana toreuma*. (a–c) Increasingly magnified views. (b) is a close up of the boxed area in (a). Acicles have wide angular ends (small arrows in (b) and (c)). Spacing between acicles varies across folia. Indexation in (a) shows that the *c*-axis lies in the plane of the folia. The big arrows in (b) point to the side edges of folia.

### 3.3 EBSD Analysis

EBSD maps obtained from radial (antimarginal) sections cut perpendicular to the shell surface, reveal that the prismatic layer is composed of large, elongated grains with varied colors (mostly green and blue) oriented at high angles to the growth surface. In contrast, the A-F layer consists of numerous narrow grains elongated parallel to the growth surface, also predominantly blue and green, occasionally purple (Figure 6a–c). This suggests that both layers share a broadly similar crystallographic orientation, although the A-F layer exhibits better co-orientation, as reflected by its more homogeneous coloration and higher MUD values compared to the prismatic layer. In the IPF maps of Figure 6a–c, MUD values are reported for the prismatic (1), transitional (2), and A-F (3) layers. The difference in MUD between the prismatic and A-F layers is significant, ranging from over 3-fold (Figure 6a,b) to more than 7-fold (Figure 6c). The PFs show generally similar orientations of the *a-* and *c-*axes in both layers. The prismatic layer exhibits a sheet texture (Figure 6b,c), occasionally transitional toward a fiber texture (Figure 6a), while the A-F layer consistently shows a well-defined sheet texture (Figures 6, and 7a,b). In all instances, the *c*-axis is nearly perpendicular to prism elongation and parallel to the folia in the A-F layer. In this layer, there is an *a*-axis (a {110} maximum) placed opposite to the folia planes, i.e., perpendicular to the folia (Figure 6a–c). The position of the {110} maxima in the prismatic layer is not so consistent. The same pattern is observed in the map of an unpolished external surface (Figure 6d), where the *c*-axis ({001} maximum) in both the transitional and A-F layers is parallel to the shell growth surface and to the direction toward the margin (Figure 6d, white arrow). The central {110} maximum indicates an *a*-axis perpendicular to the growth surface in both the A-F and, more diffusely, prismatic layers. {104} PFs provide additional information. A single set of (three) maxima appears in the transitional and A-F layers of Figure 6c, indicating a nearly single-crystal texture. In Figure 6a,b,d, two sets of maxima (∼60° apart) are observed, consistent with a {001} twin relationship. The prismatic layers in Figure 6a,b show diffuse but similar patterns.

**Figure 6:**
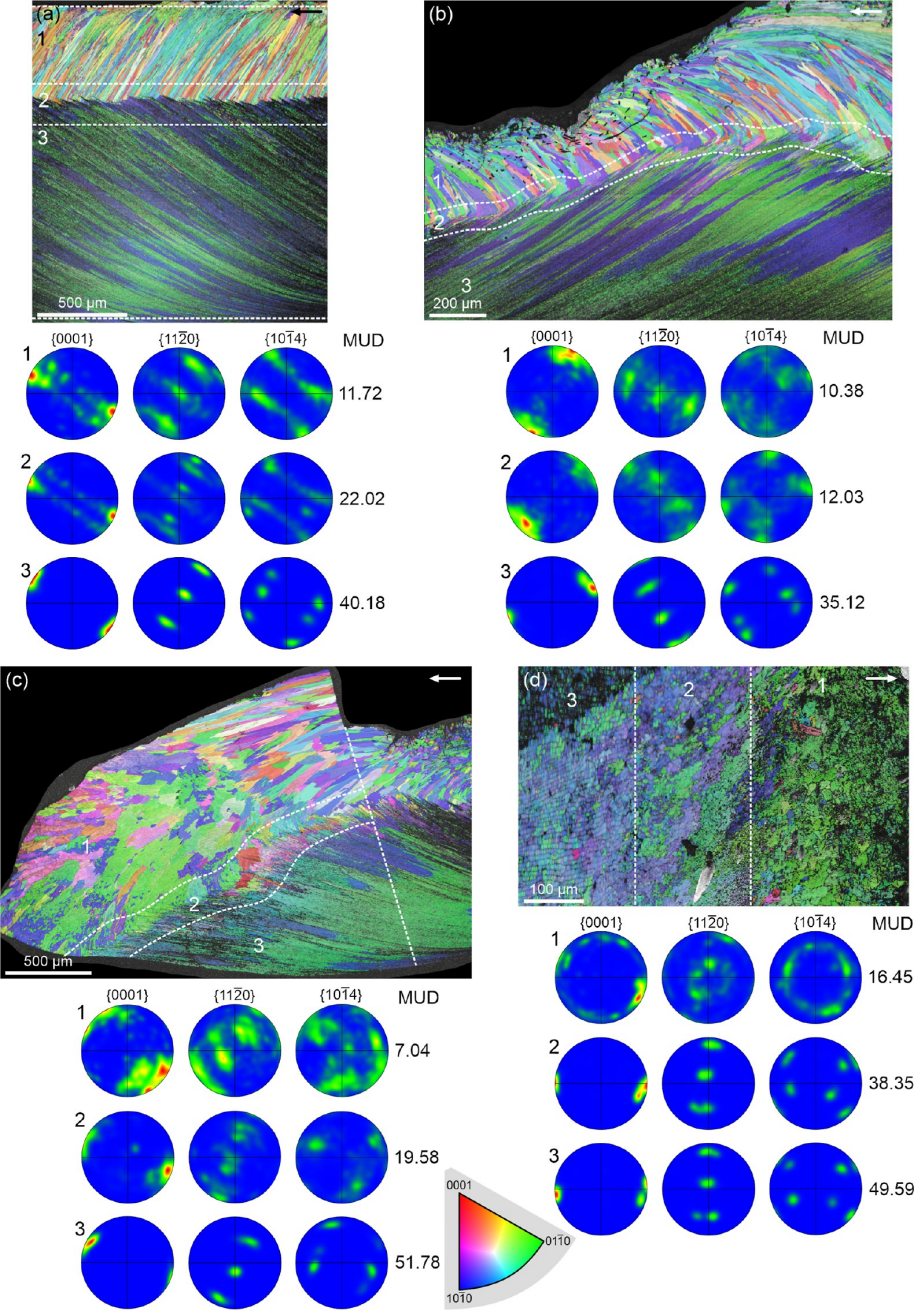
EBSD data from longitudinal sections (a–c) and an unpolished internal surface (d) of the prismatic and A-F layers. (a) *Cellana toreuma*, (b), (c) *Cellana* sp., (d) *Cellana toreuma*. The prismatic layer shows a fiber (a) or weak sheet texture (b–d), with a dominant *c*-axis maximum. The A-F layer exhibits a strong sheet texture. {104} maxima indicate a single-crystal-like texture in (c) and double-crystal-like in (a), (b), and (d). The *c-*axis is at a high angle to the long axis of the prisms and points toward the margin. According to the {001} PF of area 3 in (d), the *c*-axis is parallel to the elongation of the folia. The orientation color key applies to all maps.

**Figure 7:**
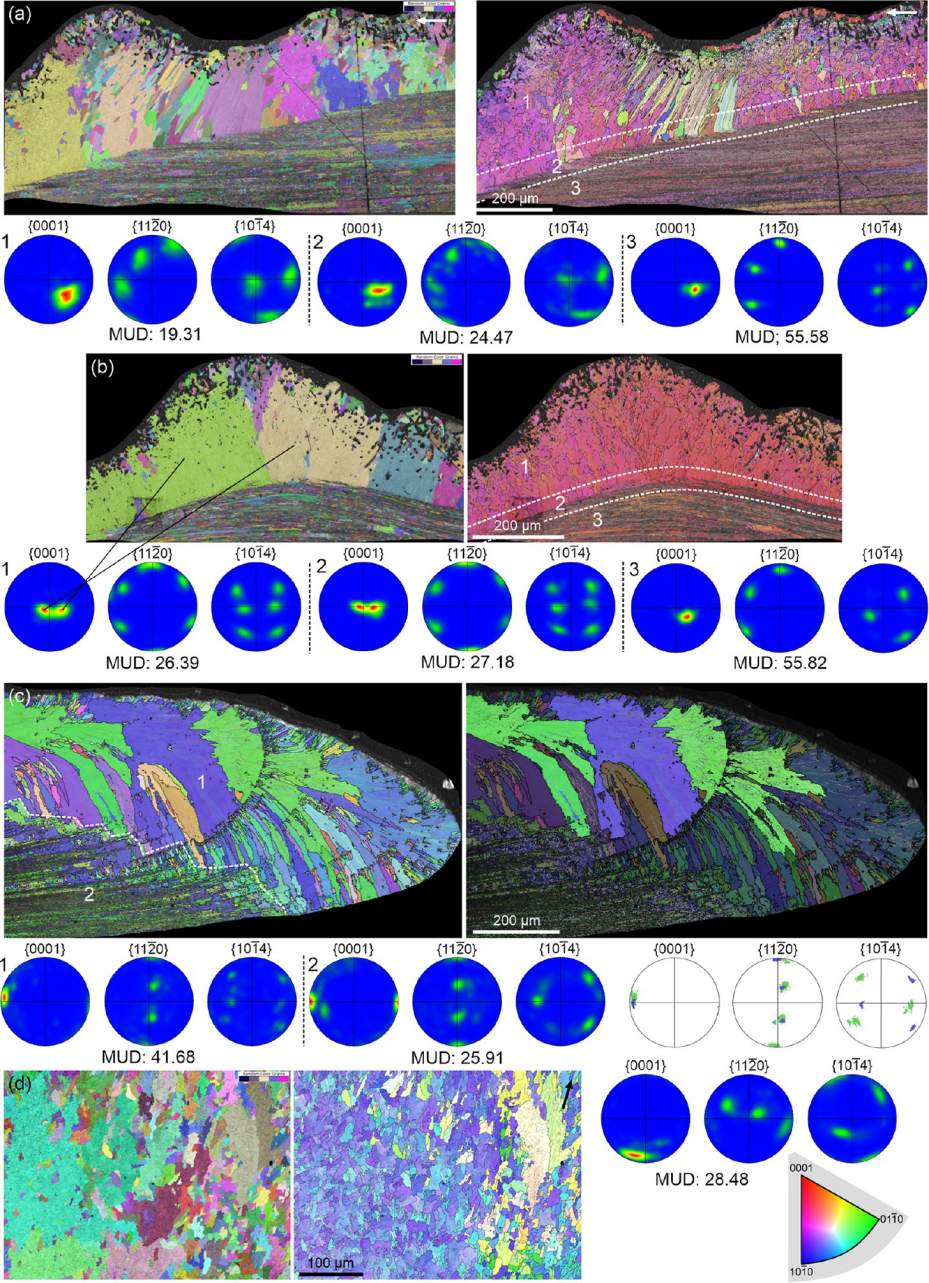
EBSD data of sections perpendicular to the shell surface through the prismatic and A-F layers (a–c) and polished horizontal section of the prismatic layer (d). (a) *Cellana toreuma*. Oblique section (∼45° to the longitudinal direction). The prismatic layer includes both slender and large prisms (GS map on the left). The texture is single-crystal-like and becomes stronger (higher MUD value) in the A-F layer. (b) *Cellana toreuma*. Commarginal section. The prismatic layer consists of several large grains (GS map on the left). The {001} PF of this layer reveals two maxima at a small distance, corresponding to two large crystallographic domains (indicated). The {104} PF contains two sets of maxima rotated around 60° (double-crystal-like texture). This texture remains in the transition layer but changes to single-crystal-like in the A-F layer, in coincidence with an increase in the texture strength (MUD values). (c) *Nacella concinna*. The prismatic layer comprises both large crystals and slender prisms. The two big grains segmented on the right have nearly coinciding {001} and {110} maxima but {104} maxima rotated by 60° (raw PFs on the right), indicating a double-crystal-like texture. The dominance of these big, co-oriented grains, provides an unusually strong texture, well above that of the A-F layer. (d) *Cellana toreuma*. The GS map reveals a general elongation of grains parallel to the growth direction. The *c*-axis aligns with the growth direction, and the texture is near single-crystal-like. The color key applies to all maps. Arrows indicate the direction toward the shell margin.

An oblique and a commarginal section are provided in Figure 7a,b, respectively. The reddish colors intensify on the IPF maps from Figure 7a,b, indicating increasing *c*-axis inclination relative to the image plane. As in radial sections, the MUD values increase from the prismatic to the A-F layer. The grain size (GS) maps (Figure 7a,b, left panels) show large grains in the prismatic layer (over several hundred μm), sometimes spanning its full thickness. These large grains either alternate with narrower prisms (Figure 7a) or dominate the layer (Figure 7b). In Figure 7b, two large grains (green and beige) dictate the crystallographic pattern and produce two close but separate {001} maxima. The {110} PFs indicate one *a*-axis parallel to the prism elongation, while the {104} PFs reveal a near single-crystal texture (Figure 7a) or a double-crystal texture with ∼60° rotation (Figure 7b). Interestingly, in Figure 7b, the A-F layer transitions to a single orientation dominated texture. A radial section from the prismatic layer of *N. concinna* also reveals a double-crystal pattern (Figure 7c), with two large crystallographic domains (blue and green) producing {104} maxima separated by ∼60° (raw {104} PF, to the right). This is the only observed case in which the MUD of the prismatic layer exceeds that of the A-F layer, due to the dominance of two large, co-oriented grains. Grain size differences within the prismatic layers are also apparent in the GS map of a polished section parallel to the growth surface (Figure 7d, left). The same map reveals a general elongation of grains in the direction toward the margin (arrow in the IPF map, Figure 7d), in coincidence with the *c*-axis direction. The PFs indicate a single-crystal-like texture.

The observed differences in MUD values between the two layers align with the calculated DADs, which consistently discriminate between the prismatic and A-F layers in all studied species (Figure S1). The A-F layers exhibit higher crystallographic order, expressed by stronger clustering of neighbor-pair misorientations around characteristic calcite twin angles near ∼60° and by the predominance of very low misorientation angles. Across all samples, the DADs show a pronounced peak at low angles (∼10°), indicating the widespread presence of low-angle boundaries and the formation of subgrain-like structures. Although the relative intensity of disorientation angles varies among species, all display the same overall trend: the A-F layer consistently shows more coherent grain-to-grain orientation relationships than the prismatic layer, in agreement with its higher texture intensity (higher MUD).

Overall, *c*-axes consistently align with the growth direction, i.e., approximately perpendicular to the long axes of prisms, and parallel to folia. In the A-F layer, a {110} maximum appears opposite to the growth surface (i.e., along the periphery of the PF in radial sections, or at the center of the PF in sections parallel to the growth surface). This is also the case for the prismatic layers in Figures 6c, and 7a–c but is unclear in Figure 6a,b, and d. With the mentioned exception of Figure 7c, the maxima in the density PFs are always better defined for the A-F layer, indicating a stronger texture (also supported by the MUD values), of either a single- or double-crystal-like type. Accordingly, texture sharpness in the prismatic layer seemingly increases with grain size.

Grain-size analysis based on EBSD maps reveals that the prismatic layer displays clear species-dependent differences in both average grain size and grain elongation. For each specimen, grain-size statistics were calculated for the full grain populations in each layer, typically ranging from 1500 to 4000 grains in the prismatic layer and 3000–6000 grains in the A-F layer. *C. toreuma* forms the coarsest and most heterogeneous prismatic microstructure, with a mean ECD of 19.85 ± 17.80 μm and extremely elongated grains reaching maximum Feret diameters of up to ∼772 μm. *N. concinna* exhibits smaller prismatic grains (mean ECD 9.62 ± 11.38 μm) but still develops occasional large prisms with maximum Feret diameters up to 481 μm. *Cellana* sp. shows the finest prismatic grains (mean ECD 7.40 ± 10.17 μm) and a moderate range of elongation, with maximum Feret diameters of 429 μm, indicating a relatively compact yet anisotropic prismatic architecture reflecting differences in grain-size distribution. Equivalent analyses show that the A-F layer is consistently finer-grained across all species. *C. toreuma* produces the coarsest foliated elements, with a mean ECD of 10.41 ± 5.56 μm and lamellae reaching up to 338 μm. *Cellana* sp. has a finer average grain size (mean ECD 6.06 ± 5.35 μm), but includes rare, exceptionally long lamellae extending to 172 μm. In contrast, *N. concinna* displays the finest and most uniform A-F structure, with a mean ECD of 6.04 ± 3.11 μm and limited elongation, reaching maximum Feret diameters of only 31 μm, indicative of a compact and tightly packed microstructure. Cropping of progressively more internal areas with an equivalent surface area shows increasing MUD values across the prismatic layer, i.e., indicating a gradual improvement in crystal order toward the A-F boundary (Figure S2).

Examination of individual prisms reveals that their typical growth orientation involves *c*-axes approximately perpendicular to their long axis (i.e., parallel to the growth surface) (Figure 8a–c). We will call these t-prisms. However, at growth interruptions, some prisms show their *c*-axes aligned along the elongation axis (l-prisms; Figure 8b–c). Following such interruptions, prisms typically return to the t-prism condition. An exception is shown in Figure 8d, where l-prisms dominate due to two growth discontinuities (red arrowheads). At these interruption fronts, new, smaller grains with an l-type orientation initiate. Only at the shell aperture (right side of large map) do a few prisms revert to t-prism type (red thick arrows). In all maps, A-F units exhibit *c*-axes parallel to the folia, except for a few in Figure 7b,d (boxed areas) where the *c*-axes are perpendicular, inherited from l-prisms.

**Figure 8:**
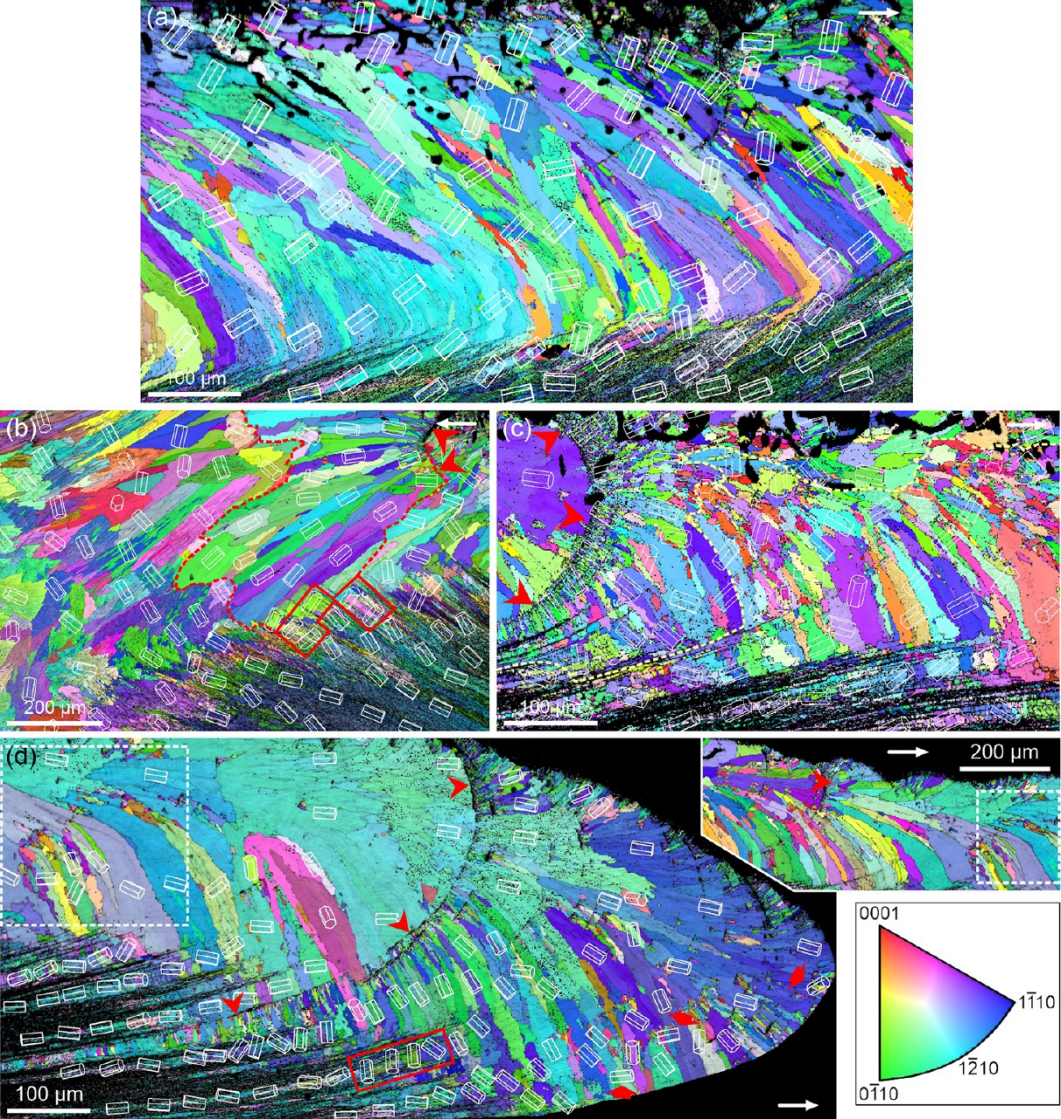
Prism orientations in IPF maps. (a) *Cellana* sp. Prisms mostly orient their *c*-axis at a high angle to their long axis (t-prisms), retained within the A-F layer. (b) *Cellana* sp. Following a growth discontinuity (red arrowheads), the prisms have their *c*-axis oriented parallel to their long axis (l-prisms). After a certain growth span, the prisms revert to t-prisms. The boundary between l- and t-prisms is indicated with dashed red line. Some A-F units retaining the l-condition are outlined in red. (c) *Nacella cocinna*. As in (b), after a growth discontinuity (red arrowheads), prisms are of the l-type. Subsequently, prisms revert to the t-type. The dashed line marks the transition. (d) *Nacella concinna* (same specimen as in Figure 7c). Growth is dominated by l-type prisms. Their persistence may be attributed to two shell growth interruptions. The first interruption is indicated (red arrowheads) in the inset, and the second interruption is indicated (red arrowheads) on the big map on the left. Only at the shell margin do some prisms become t-prisms (red arrows). Some A-F units with an l-condition are red-framed. The two dashed white boxes in both the inset and the large map overlap with each other. The color key applies to all maps. White arrows point toward the shell margin.

T-prisms can curve with growth to match the changing orientation of the growth lines (Figure 9). Concomitantly, the *c*-axes reorient to remain roughly perpendicular to prism elongation and parallel to the growth lines. Growth trajectories plotted on the PFs for the selected crystals of Figure 9b,c, show pole migration and internal misorientation gradients reflecting gradual lattice rotation involving the *c*- and *a*-axes. This behavior is absent in l-prisms, whose axes remain stationary regardless of orientation to the growth surface, as deduced from the orientations of the unit cells and uniform colors in Figure 8d, and from the high pole clustering for the two big grains selected in Figure 7c. KAM maps show that t-prisms contain many internal low-angle boundaries (Figure S3a–d), possibly related to organic-molecule-induced dislocations. Figure S3d is the KAM map for Figure 8b, where we have marked the approximate boundary between t- and l-prisms. This boundary, plotted in Figure S3d, is not exactly coincident, but close to that between the areas with scarce and abundant misorientation boundaries. In contrast, the KAM map in Figure S3e (mostly l-prisms; Figure 8d) shows a general absence of internal boundaries.

**Figure 9:**
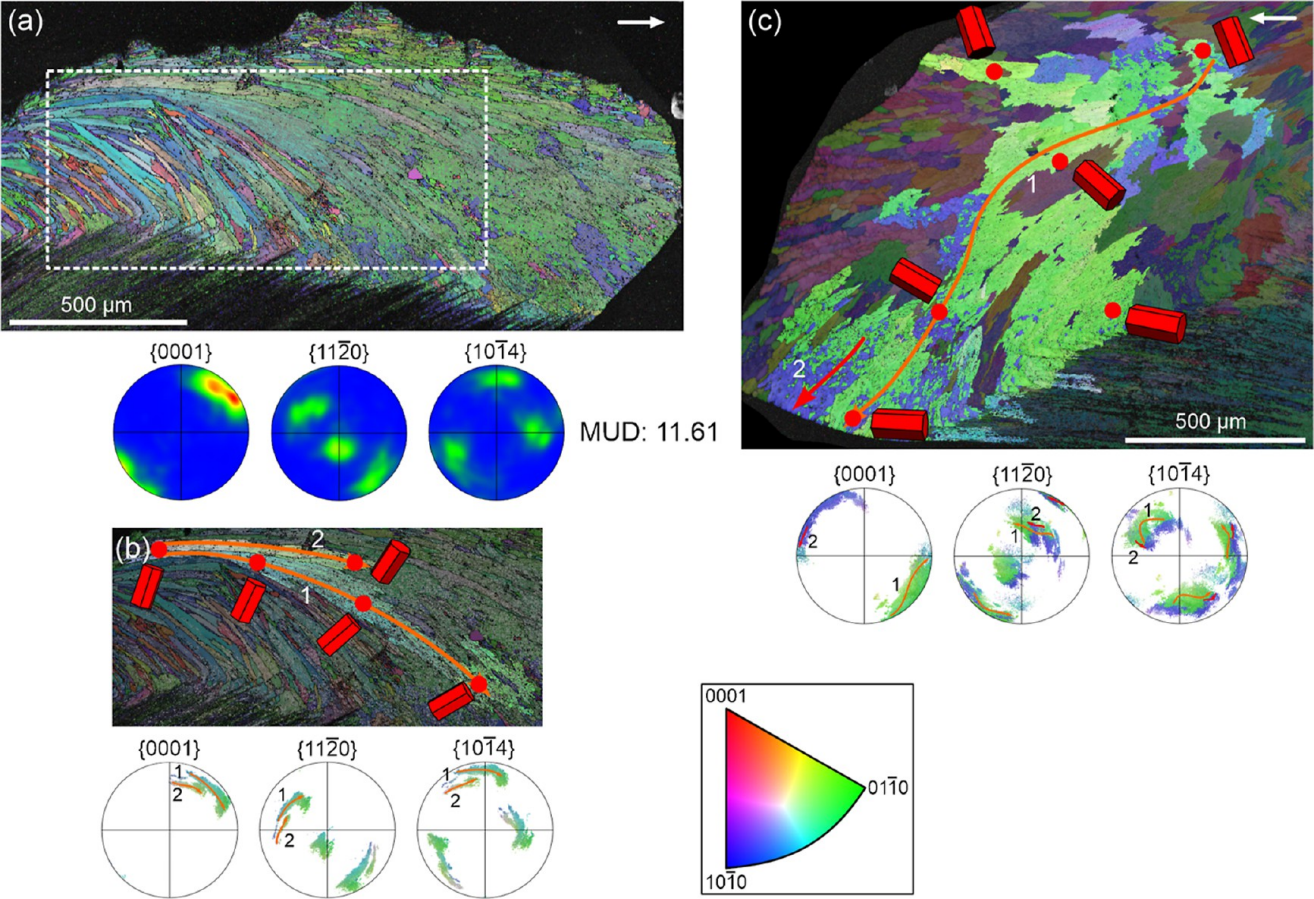
Orientation changes in t-prisms. (a–c) *Cellana* sp.. Sections perpendicular to the shell surface. (a) The *c*-axis is nearly perpendicular to the elongation of prisms (i.e., t-prisms). Elongation of PF maxima results from prism curvature. (b) Change in orientation in a prism from (a) (boxed). Unit cells show the *c*-axis shift. The growth trajectories depicted in the raw PFs show the changes in the orientation of axes with growth. (c) The changes in the crystallographic axes of the large, selected grain are indicated by both the unit cells and the growth trajectories on the raw PFs. In both (b,c), the *c*-axes remain roughly parallel to the growth surfaces. The color key applies to all maps. White arrows indicate the direction toward the shell margin.

The boundary between the prismatic and A-F layers is characteristically stepped, with prisms partitioning into transverse segments forming a staggered front. This is evident on the IPF maps of Figure 10. Crystal domain widths may fluctuate at these interfaces, until the segments are fully incorporated into the A-F layer (Figure 10). Some prismatic grains appear interrupted at the transition (Figure 10b,d,e, thick black arrows), while other prismatic segments emerge (Figure 10b−e, blue and red arrows). One polished section parallel to the growth surface shows a prismatic crystallographic domain extending directly into the A-F layer (Figure 10f, thick black arrow), suggesting localized inheritance.

**Figure 10:**
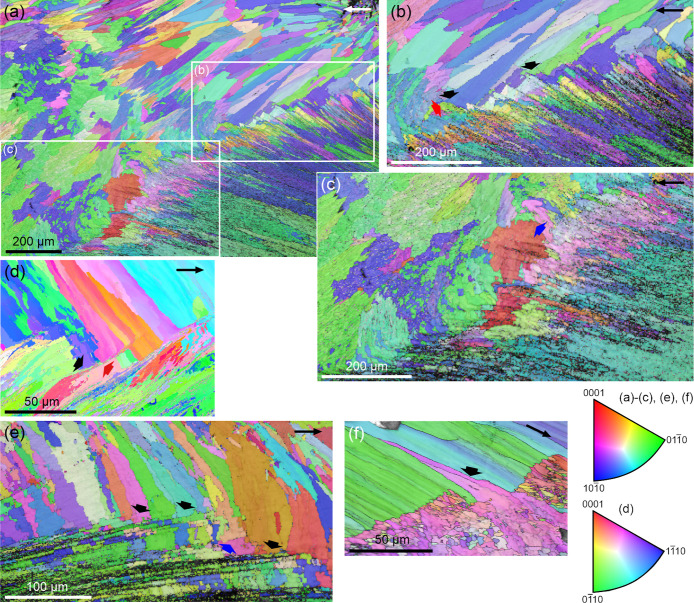
Prism transformation at the boundary with the A-F layer. (a–e) Horizontal partition of prisms. (a–c). *Cellana* sp. (d) *Cellana tramoserica*. (e) *Nacella concinna*. Thick black arrows indicate prisms that stop at the boundary with the A-F layer. Red or blue arrows point to prismatic segments that seemingly emerge at the transition. (f) *Cellana toreuma*. Polished section parallel to the growth surface. The thick arrow points to a prism continuing into an A-F unit. The color key for each map is indicated. Long arrows on the top right corners indicate the direction toward the shell margin.

EBSD maps from intact inner surfaces of the A-F layer consistently show a {001} maximum distribution, indicating that *c*-axis is oriented parallel, although not strictly, to the elongation of the folia (Figures 6d, area 3, and 11a–c). Polished inner surfaces reveal slight misalignments of *c*-axes between adjacent folia typically <4° (Figure 11d). As commented on above, there is a central maximum on the {110} PF, indicating that the *a*-axis is perpendicular to the growth surface. This is consistent with results from transversal sections (Figure 11e,f). Based on the PFs in Figure 11b, acicles generally align (though not perfectly) along a <100> direction, though divergence is evident in Figure 11c. From the IPF map of Figure 11g (left), grains (domains) were segmented using a 10° misorientation threshold (Figure 11g, GOS map, to the right). Domains have low internal misorientations (maximum 6.75°), contain from a few to several tens of acicles and extend across several superposed folia. Misorientation profiles confirm minimal differences between acicles within domains (Figure 11g, bottom). Figures 11b, and S4 reveal two distinct maxima on the {001} PF and two related sets of {104} maxima rotated ∼60°, indicating a twinning relationship. The areas that correspond to each set are numbered in Figure 11b and color-coded in Figure S4. In Figure S4a–c, the misorientations across the boundary between the two big crystalline domains are ∼60° (misorientation profiles). A domain in green in Figure 11g (red arrow on the IPF map and misorientation profile 1) is misoriented by ∼60° from its neighbors. Its even color indicates rotation mainly around the *c*-axis. Another type of relationship is provided by a few crystalline domains in red/dark orange on the IPF map of Figure 11g, left, which also exhibit high, though different misorientations (80–90°), also shown in the misorientation profiles. Their red colors indicate that the *c*-axes are at high orientations with respect to the growth surface. Clusters of this kind belong to the red-orange central maxima in the {001} PF of Figure 11a (blue arrows).

**Figure 11:**
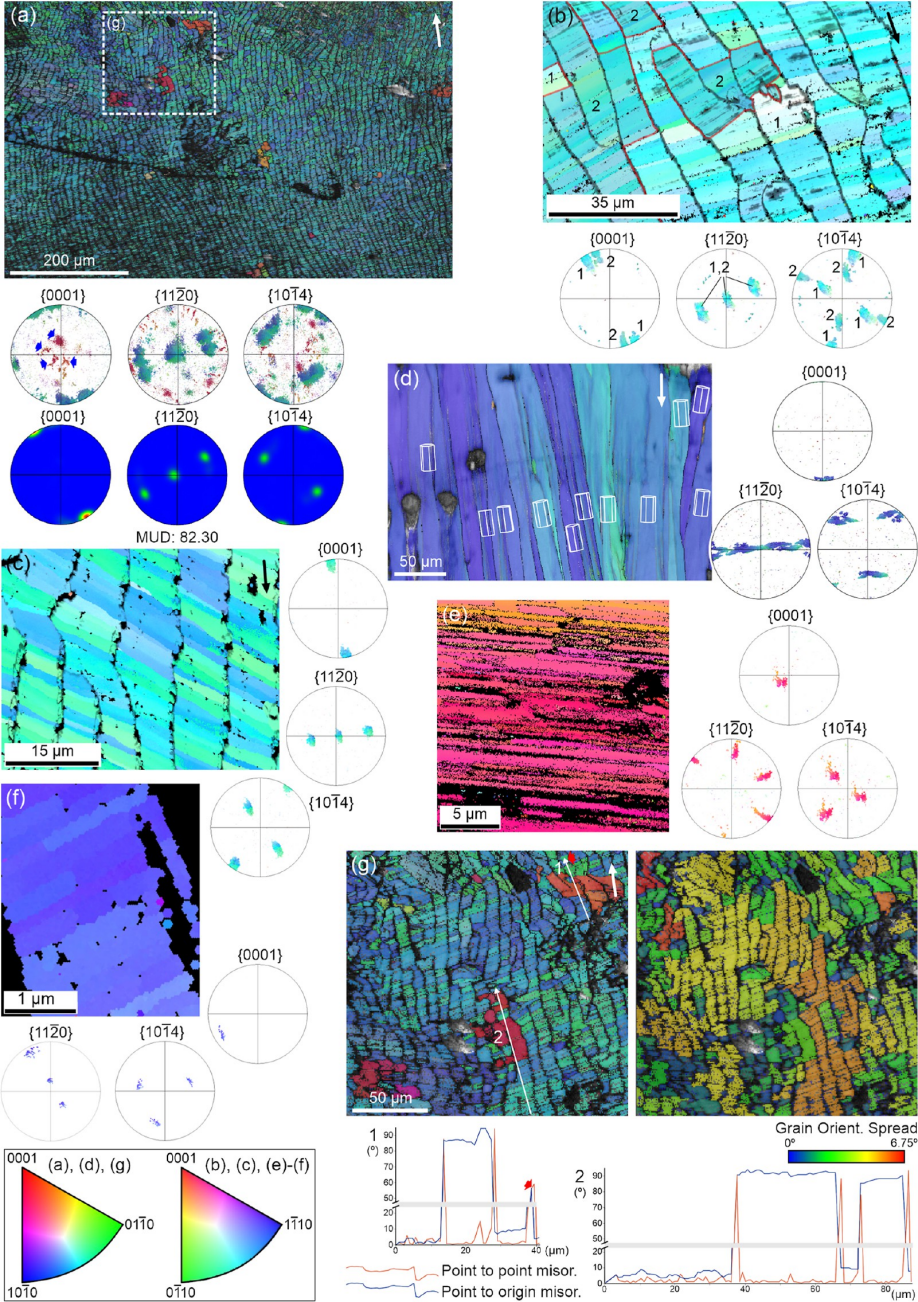
EBSD analysis of the A-F layer of *Cellana toreuma* *.* (a–c) IPF maps of unpolished growth surfaces. The *c*-axis is parallel to the elongation of the folia, and there is always a pole maximum at the center of the {110} PF, indicating that an *a*-axis is perpendicular to the growth surface. A single-crystal-like texture is observed in (a) and (c), and a double-crystal-like texture in (b), where there are two sets of {104} maxima. In (a), the positions of the {001} maxima for the red/orange domains are indicated with blue arrows in the {001} PF. In (b), the areas that contribute to each maximum on the {104} PF have been delineated and indicated with numbers (see also Figure S4a); areas numbered 2 have also been slightly shadowed. In (b) and (c), acicles in slightly different colors suggest slight misorientations. In (c), some acicles in even colors extend across superposed folia. (d) Section parallel to the folia. The *c*-axis is parallel to the elongation of the folia, as indicated also by the cell lattices, with some spread due to the proximity of the boundary with the prismatic layer. (e) Section transversal to the folia. The *c*-axis is oriented parallel to the folia, meaning that it is nearly perpendicular to the projection plane, while a {110} maximum, corresponding to an *a*-axis, is aligned perpendicular to the folia. (f) *t*-EBSD map of an FIB section transversal to the folia planes. The distribution of maxima with respect to folia is as in (e), although the *c*-axis is more parallel to the sectioning plane. (g) Cropped area of (a) (boxed). Two misorientation plots have been traced along particular folia on the IPF map (left). The misorientation between acicles is low (<5°), except at the contacts with the red domains, where they rise to 80–90°. In profile 1, the final peak at ∼60° corresponds to a green domain on the IPF map at the end of transect 1 (red arrows on both the IPF map and profile 1). Individual grains are delineated on the GOS map (right), many of them extending across folia. Misorientation within grains does not exceed 6.75°. The color key for each map is indicated. Arrows in the upper right corners of the maps in (a)–(d) and (g) indicate the direction toward the shell margin.

### 3.4 Mechanical Properties

The mechanical properties of the limpet shells were quantified using two complementary nanoindentation approaches (Figure S5). First, surface nanoindentation was conducted on the internal surfaces of both the external (prismatic) and internal (A-F) layers of *N. concinna* under dry and wet conditions, directly probing the mechanical response of each layer along its growth surface (Figure S5a). The prismatic layer consistently exhibits higher hardness and reduced modulus than the A-F layer. Under dry conditions, the prismatic layer reaches a hardness of 1.9 ± 0.5 GPa and a reduced modulus of 39.6 ± 3.2 GPa, whereas measurements under wet conditions show a marked decline in both parameters (0.4 ± 0.1 GPa and 14.7 ± 1.4 GPa, respectively). The A-F layer displays substantially lower mechanical resistance in the dry state, with a hardness of 1.6 ± 0.4 GPa and a reduced modulus of ∼21 ± 3.4 GPa. Hydration of the A-F layer further decreases these values (0.5 ± 0.1 GPa and 4.4 ± 0.6 GPa), consistent with the dominant role of mineral–organic interfaces in accommodating deformation within this layer. For comparison, surface nanoindentation of hydrothermal calcite yields reduced modulus values (∼40 ± 3.5 GPa) similar to the stiffest prismatic regions, although its hardness remains distinctly lower (∼0.6 ± 0.1 GPa). These results indicate that the dry prismatic region approaches the intrinsic stiffness of abiogenic calcite despite its biogenic origin.

In the second approach, nanoindentation was performed on polished cross sections of both shell layers of *C. toreuma* under dry conditions and on geological calcite (Figure S5b), which exhibits a hardness of 2.9 ± 0.1 GPa and a reduced modulus of 66.5 ± 1.3 GPa. The prismatic layer of *C. toreuma* displays nearly identical values (3.0 ± 0.1 GPa and 68.0 ± 2.1 GPa, respectively), confirming that the large prisms behave as densely packed, mechanically robust calcite crystals. In contrast, the A-F layer shows lower hardness (2.7 ± 0.3 GPa) and a distinctly reduced modulus (60.0 ± 2.6 GPa).

These mechanical measurements demonstrate that the two shell layers fulfill fundamentally different structural roles, reflected in their contrasting mechanical regimes. The outer prismatic layer shows markedly higher hardness and stiffness, while the inner A-F layer occupies a lower-stiffness regime.

## 4 Discussion

MacClintock^14^ originally classified the Nacellidae into four microstructural groups: group 11 included *Nacella*, and groups 12–14 encompassed *Cellana*, which displayed only minor microstructural variations. This classification was later reevaluated by Fuchigami and Sasaki,^15^ who merged groups 12, 13, and, tentatively, 14 into a single group. All these species share the same prismatic + A-F outer shell layers analyzed in the present study (Figure 12).

**Figure 12:**
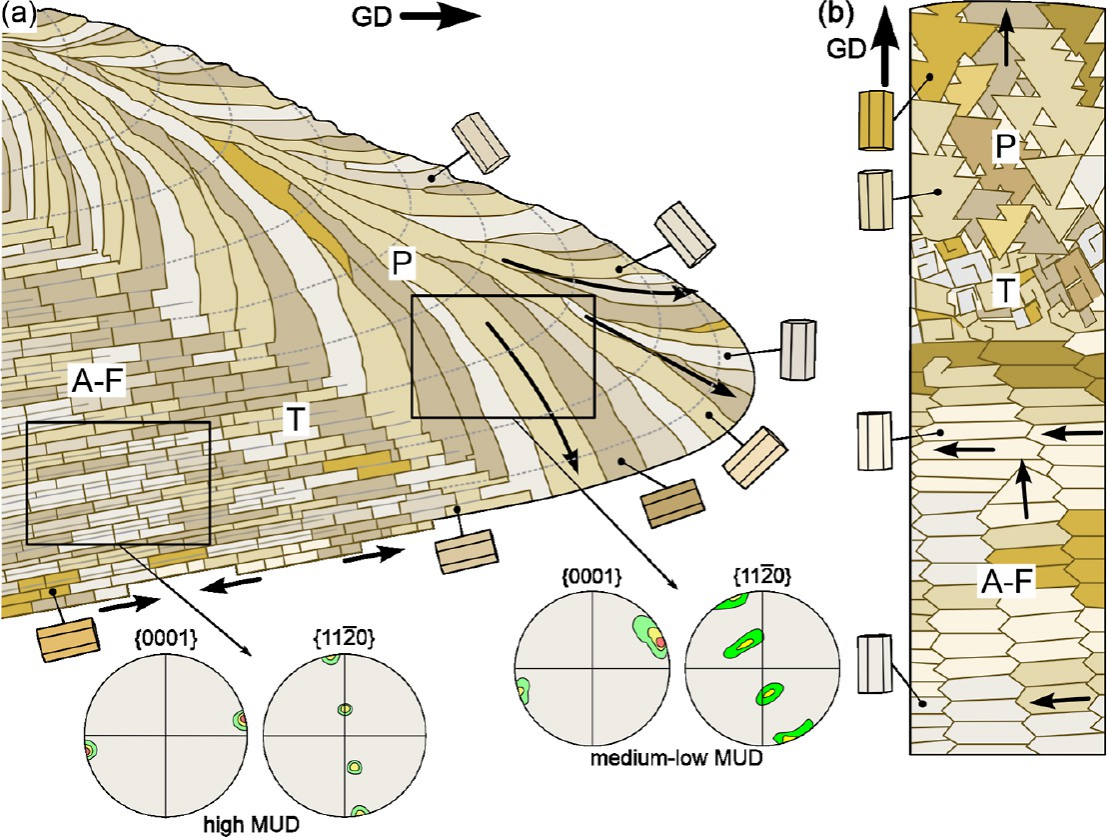
Diagram illustrating the distribution of calcitic shell layers of Nacellidae. (a) Radial section. The shell consists of an outer prismatic layer, a transitional zone, and an internal A-F layer. The prismatic layer is composed of prisms that elongate at a high angle to the growth margin (long arrows). However, their *c*-axes are oriented at a high angle to their long axes, as indicated by the depicted cell lattices and by the position of the maxima in the {001} pole figure. The prisms also orient one of their *a*-axes perpendicular to the shell growth surface ({110} maxima). This orientation is inherited by the A-F layer, although its crystallographic texture becomes stronger (higher MUD values and reduced pole figure maxima). In the transition zone, the prisms become horizontally sliced, and these slices split further within the A-F layer. (b) Plan view of the shell growth surface showing the prismatic, transitional and A-F layers. Prisms elongate in the direction toward the margin. Different colors denote distinct crystallographic orientations but are also intended to distinguish better the different elements. A-F: A-F layer, GD: growth direction, P: prismatic layer, T: transition layer. Thin arrows indicate local growth directions, whereas thick arrows indicate the overall growth direction of the shell margin.

The prismatic and A-F layers in nacellids share a common crystallographic texture, characterized by a {001} maximum aligned with the radial (longitudinal) growth direction and a {110} maximum oriented toward the shell internal surface (Figure 12a). This configuration implies that the *c*-axis lies within the growth surface, pointing toward the shell margin, while one *a*-axis is perpendicular to the growth surface. The prismatic layer may display either a single- or double-crystal-like texture based on the distribution of {104} maxima, independent of grain size or number. In the double-crystal-like case, the {001} and {110} maxima of both domains are closely aligned, while their {104} maxima differ by ∼60°, suggesting a potential {001} twin relationship between dominant grain orientations. Slight mismatches between maxima are expected due to common intragrain misorientations. This twin-like texture is also seen in the A-F layer (Figures 11b, and S4), probably inherited from the prismatic layer. Except for rare cases (e.g., Figure 7c), the crystallographic texture is markedly stronger in the A-F layer (Figure 12a), suggesting progressive selection of energetically favorable orientations during growth. The DADs further support this interpretation. In all species, the prismatic layer shows neighbor-pair misorientation distributions close to the Mackenzie curve, indicative of weakly correlated grain orientations and a predominance of high-angle boundaries. Conversely, the A-F layer exhibits strong enrichment in low-angle misorientations (∼10°) together with distinct peaks near 60°, revealing that adjacent crystals tend to share similar orientations and that coherent crystallographic domains develop within individual folia.

While prisms with a *c*-axis transverse to their elongation (t-prisms) are typical, prisms with the *c*-axis longitudinal (l-prisms) appear following growth interruptions that disrupt continuity with the previously secreted prisms. After a certain time lapse, new prisms are of the t-type. T-prisms can reorient their *c*-axis during growth to accommodate to the changes in the orientation of the growth front (Figures 9, and 12a). This flexibility is enabled by intraprismatic low-angle boundaries (Figure S3), arising from gradual lattice rotation. These subgrain structures, mediated by organic molecules, allow preservation of crystallographic continuity despite changes in growth direction.

Grain-size statistics complement the crystallographic observations by demonstrating clear species-dependent microstructural differences. The prismatic layer differs markedly across taxa. *C. toreuma* exhibits the largest and most elongated prisms, *Cellana* sp. shows the finest and least elongated prisms, and *N. concinna* shows intermediate values with occasional very large domains. In contrast, interspecies differences in the A-F layer are more limited.

These microstructural distinctions are directly reflected in the mechanical measurements. The prismatic layer consistently occupies a narrow high-stiffness regime irrespective of species, an architecture well suited to resist external mechanical challenges such as sediment abrasion, and impact loading including potential predatory attack. In contrast, the A-F layer exhibits strong mechanical anisotropy, with substantial decreases in hardness and reduced modulus depending on loading direction, reflecting its laminated architecture and higher density of internal organic interfaces. It is important to emphasize the drastic decline in both parameters when the samples are tested wet, reflecting conditions closer to their native state. Together, the two layers represent a coupled protective system: a stiff exterior and a more compliant interior.

The observed sheet texture and, particularly, the distribution of crystallographic axes of the prismatic layer has never been found in prismatic materials. Prismatic and fibrous calcitic microstructures so far studied in molluscs always exhibit a fiber texture with the *c*-axis of calcite as the fiber axis and parallel to the prism elongation, as seen in various bivalves,^18–21^ gastropods,^22^ and cephalopods.^7,23^ Outside molluscs, this has also been reported for brachiopods,^24,25^ foraminifers,^26,27^ and bird eggshell.^28^ Such an orientation is typically interpreted as the result of competitive growth between crystals nucleated on an organic matrix and sharing a common growth front. Selection favors grains with optimal orientations, that is, with the fastest growth axis, the *c*-axis in calcite, typically oriented perpendicular to the substrate.^29,30^ Competition leads to grain number reduction, survivor grain coarsening, and texture sharpening.

Recently, we have identified two calcite microstructures in gastropods where one {104} face aligns parallel to the growth front: the crossed-foliated layer of Patellidae,^31^ and the granular-prismatic layer in the calcitic gastropod *Epitonium* (unpublished work, Granada and Cracovia, 2024-2025). We interpreted these instances as due to nucleation of crystals on {104} onto an organic template (the mantle). Similarly, the nacellid prismatic layer growth, with a {110} face oriented parallel to the mantle surface, suggests a unique biological interaction guiding calcite crystallization.

Still, oriented nucleation on {110} faces alone would primarily yield a fiber texture with the *a*-axis as the fiber axis. The consistent orientation of the *c*-axis in the radial direction of nacellids implies an additional mechanism, likely orientation selection via crystal competition on the shell growth surface. In nacellids, new prismatic elements nucleate at the shell margin and grow inward (Figure 12a). As they thicken, a secondary competition likely takes place within the growth surface, favoring grains whose *c*-axes (which is the fastest growth axis of calcite, and is contained within this surface) align toward the margin (Figure 12b). This interpretation is supported by SEM observations of the growth surfaces of the prismatic layer, which show that crystals tend to elongate preferentially in the direction toward the margin, sometimes producing subsidiary rhombohedral elements (Figures 2f,h, and 7d). EBSD sections through the prismatic layer show that the number of prisms decreases with depth (Figures 6a,b, and 8a,c,d), and that the texture strength increases with growth (Figure S2). All these features are hallmarks of competitive growth. However, additional data are needed to verify this hypothesis. Cases like the nearly single-crystal-like textures of Figure 7a,b suggest that epitaxial nucleation also contributes to orientation coherence. Without epitaxy, the occurrence of the two possible rhombohedral orientations would be equiprobable. All in all, the crystallographic distributions observed thus imply an interplay of biologically guided nucleation, refined by selection via competitive growth and mutual epitaxy between prismatic grains.

At the prismatic and A-F layer boundary, prisms become transversely segmented and are incorporated into the A-F layer in a conveyor-belt-like manner (Figure 12a). We interpret segmentation as resulting from slicing by thin organic interfaces parallel to the growth surface. As crystal domains traverse these interfaces, their widths change or even become halted entirely (Figures 10a–e, and 12a). Subsequently, the slices further subdivide into folia (Figures 10a–e, and 12a) and become fully incorporated into the A-F layer. Each segment finally transforms into coherent folia sets (Figures 11g, GOS map, and Figure 12a). Adjacent domains misoriented by ∼60°, likely {001} twins (Figures 11b, and S4), appear to derive from the prismatic structure. Another case of transmission of the crystallography from the prismatic to the A-F layer is that of segments derived from l-prisms (with the *c*-axis transversal to the growth surface; Figure 8b,d), which originate sets of acicles whose *c*-axes retain that orientation (Figure 11a,g).

The stronger texture of the A-F layer, compared to the prismatic layer, likely results from multiple mechanisms: (1) competition among sets of acicles within each folium (Figure 4d–f), favoring those aligned at a higher angle with folia elongation; (2) the flatter, more continuous growth surfaces of folia providing better interfacing with the organic template; and (3) epitaxial emergence of new folia from existing ones (Figures 4d–g, and 11g), transmitting crystallographic orientation across layers. Together, these promote the development of a highly ordered material.

Individual folia elongate preferentially along the *c*-axis (Figures 6d, 11a–d, and 12b), which is already radially aligned in the prismatic layer. One may hypothesize whether the special crystallographic organization of the prismatic layer is a strategy to achieve a convenient crystallographic orientation of the folia. While speculative, a *c*-axis (which is usually a preferred growth direction in calcite) perpendicular to the growth surface would hinder folia extension. The observed exfoliation patterns (Figure 4a,g,h,k,l) reveal extreme folia and acicle lengths, high splitting capacity, and remarkable flexibility. This flexible behavior is consistent with the reduced mechanical-property values measured for the A–F layer under wet conditions (Figure S5), which approximate the hydrated state of the shell in living animals. All this suggests that the A-F layer functions as a high-performance biocomposite constructed from weak basic components (calcite and organics). This sophisticated architecture may be rooted in the design of the prismatic precursor layer.

While the prismatic layer resembles the noncolumnar prismatic structure found in other molluscs (see above), the A-F layer is morphologically unique in that it comprises extensive radial folia subdivided into transverse acicular units. In conventional foliated microstructures (e.g., bivalves), the laths (analogous to the nacellid acicles) are considerably wider (typically 2–5 μm) but of similar thickness (200–250 nm thick).^32^ Their arrangement ranges from independent units to, rarely, extensive folia (e.g.,^1,2,32^ ). Moreover, bivalve folia tend to orient roughly parallel to the shell margin.

We also examined the foliated layer of a fragmentary specimen of the patellogastropod *E. vitrea* (family Lepetopsidae, superfamily Lottioidea), which also secretes a foliated layer.^16^ This material is composed of laths several tens of microns wide, with pointed or truncated arrow-like terminations, arranged into well-organized folia (Figure S6a–c). Its morphology closely resembles the foliated calcite found in the bivalve *Anomia ephippium*.^2,32^ PFs show that the *c*-axes are contained within the lath planes and parallel to their elongation (Figure S6d,e), while the other crystallographic axes are poorly co-oriented. Thus, the foliated microstructure of *Eulepetopsis* is both morphologically and crystallographically distinct from the A-F microstructure of the Nacellidae. *Eulepetopsis* belongs to the Lottioidea, a different superfamily from the Patelloidea, where the Nacellidae resides, i.e. the two families are unrelated.

In bivalves, lath growth along high-angle directions relative to the *c*-axis is thought to be stabilized by organic molecules.^33–35^ Outside molluscs, foliated materials are found in stenolaemate bryozoans^36^ and inarticulate brachiopods,^25^ both with *c*-axes aligned in-plane with laths. However, neither group develops extensive folia, and only stenolaemates exhibit a true sheet texture.

Overall, the prismatic and acicular-foliated layers of nacellids represent a singular morphological-crystallographic innovation, endowing each layer with different mechanical functions that appear unparalleled among molluscs, and, indeed, across metazoans. The integration of stiff prisms with compliant, deformation-accommodating folia provides a unique strategy for building strong, resilient, and mechanically efficient calcite-based shells.

## Supplementary Material



## Notes

Disorientation angle distributions for the prismatic and A–F layers of *Cellana toreuma*, *Cellana* sp., and *Nacella concinna;* MUD values for progressively more internal areas of the prismatic layers of *C. toreuma*, and *C. tramoserica*; KAM maps corresponding to IPF maps of *Cellana* sp., and *N. concinna*; EBSD maps on the unpolished growth surfaces of *Cellana* toreuma, and *C. testudinaria* revealing a double crystal-like texture; SEM and EBSD data on the foliated microstructure of *E. vitrea* (PDF)
