# Supplementary material for: Calcite Tailoring by Limpets: The Prismatic and Acicular-Foliated Microstructures of Members of The Nacellidae
Source: Cryst Growth Des. 2026 Jan 15;26(3):1160–77. doi: 10.1021/acs.cgd.5c01236 (PMC12879962; doi:10.1021/acs.cgd.5c01236)
Supplement: Supplementary file 1 [file cg5c01236_si_001.pdf]

Calcite Tailoring by Limpets: The Prismatic and Acicular-Foliated Microstructures of Members of the Nacellidae

Katarzyna Berent [a], Marta Gajewska [a], Antonio G. Checa [b,*]

[a] *Academic Centre for Materials and Nanotechnology, AGH University of Science and Technology, 30 Kawiory Street, Krakow, 30-059, Poland*

[b] *Departamento de Estratigrafía y Paleontología, Universidad de Granada, s/n Fuentenueva Street n/n, Granada, 18071, Spain*

Supplementary material

Figures S1 to S6

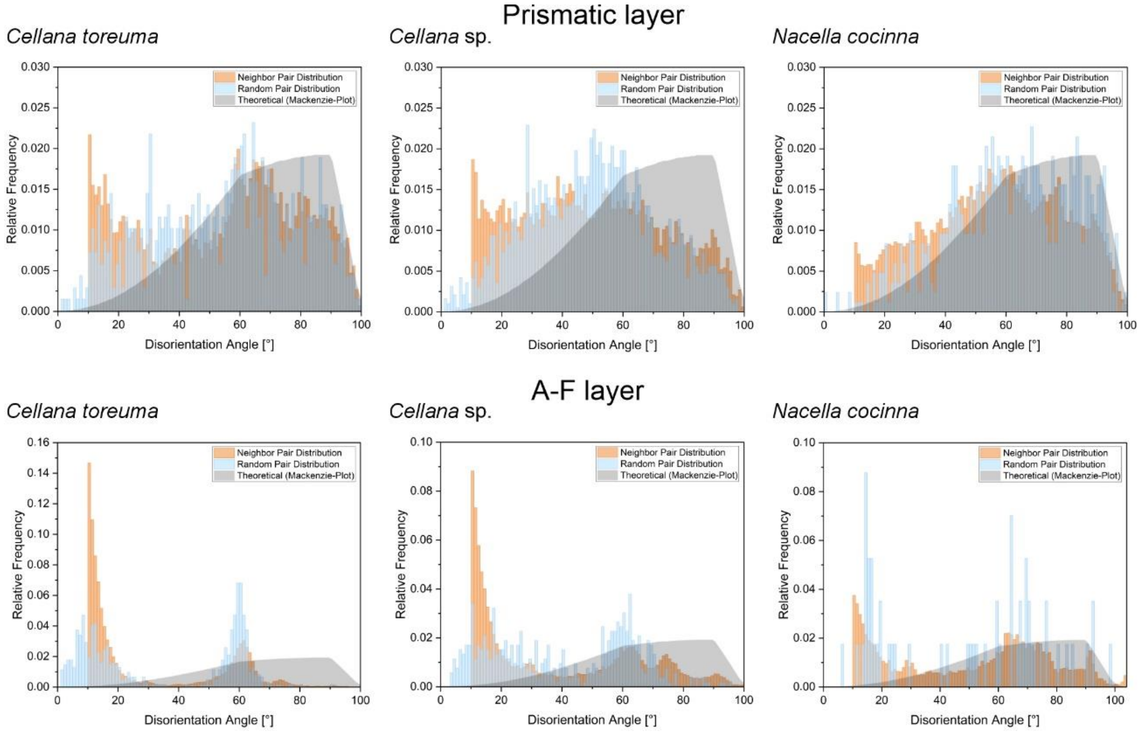

Figure S1. Disorientation angle distributions (DADs) for the prismatic (top row) and acicular–foliated (A–F) layers (bottom row) of *Cellana toreuma*, *Cellana sp*., and *Nacella concinna*. For each specimen, two distributions are shown: (i) the neighbor-pair distribution (orange), representing misorientation angles between directly adjacent grains; and (ii) the random-pair distribution (blue), calculated from randomly selected grain pairs within the same EBSD map. These are compared with the theoretical distribution for randomly oriented crystals (gray curve), given by the Mackenzie distribution for calcite symmetry.

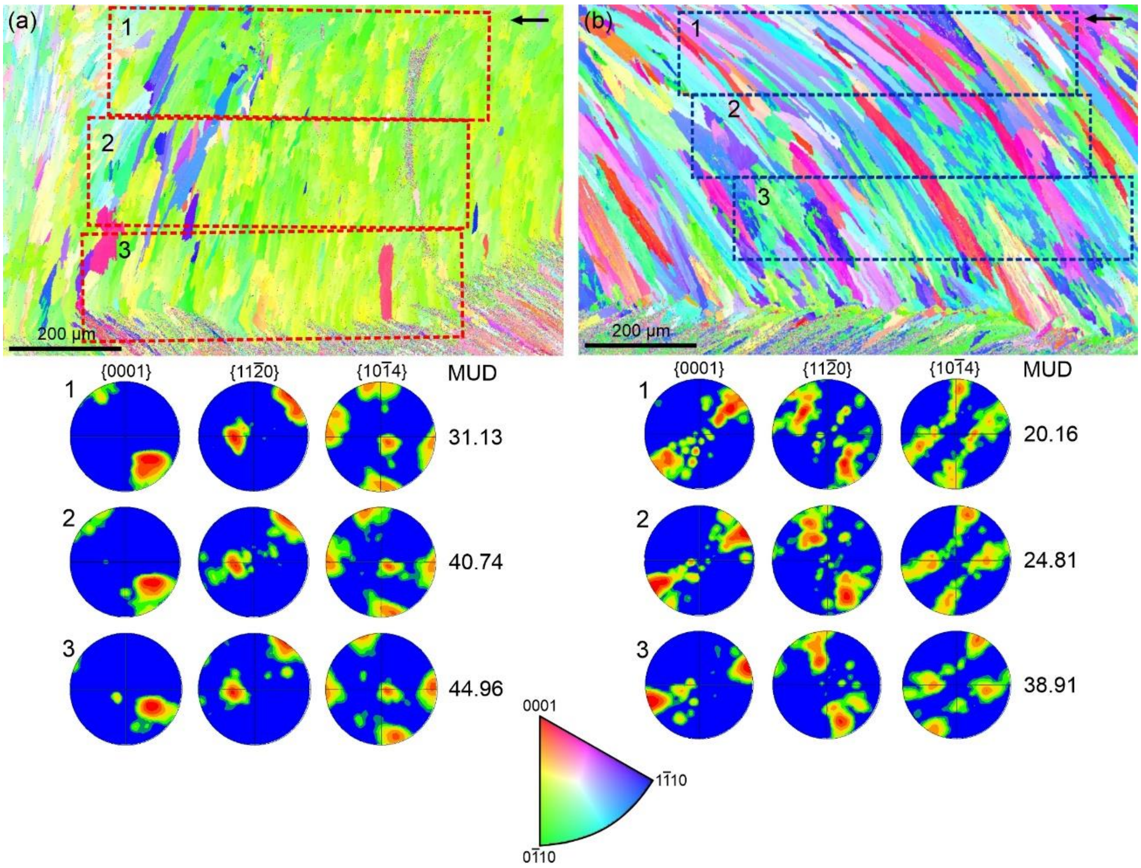

Figure S2. IPF maps, pole figures, and MUD values for progressively more internal regions of the prismatic layer of (a) *Cellana toreuma*. (b) *Cellana tramoserica*.

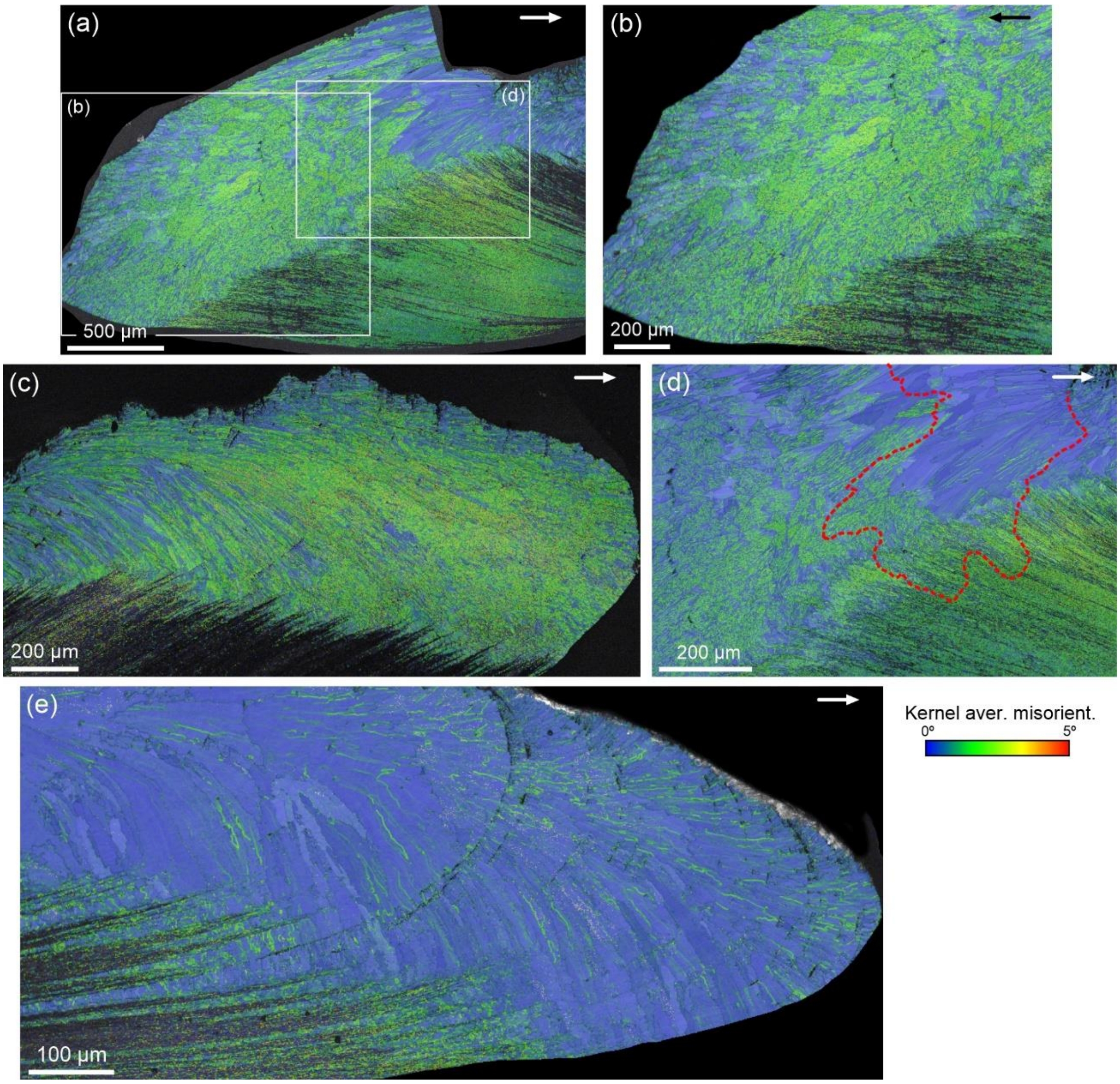

Figure S3. KAM maps corresponding to the IPF maps shown in (a) Figure 6c, (b) Figure 9c, (c) Figure 9a, (d) Figure 8b, and (e) Figure 8d. (a)-(c) *Cellana* sp., (e) *Nacella concinna*. Maps (b) and (d) are cropped areas of (a) (boxed regions).

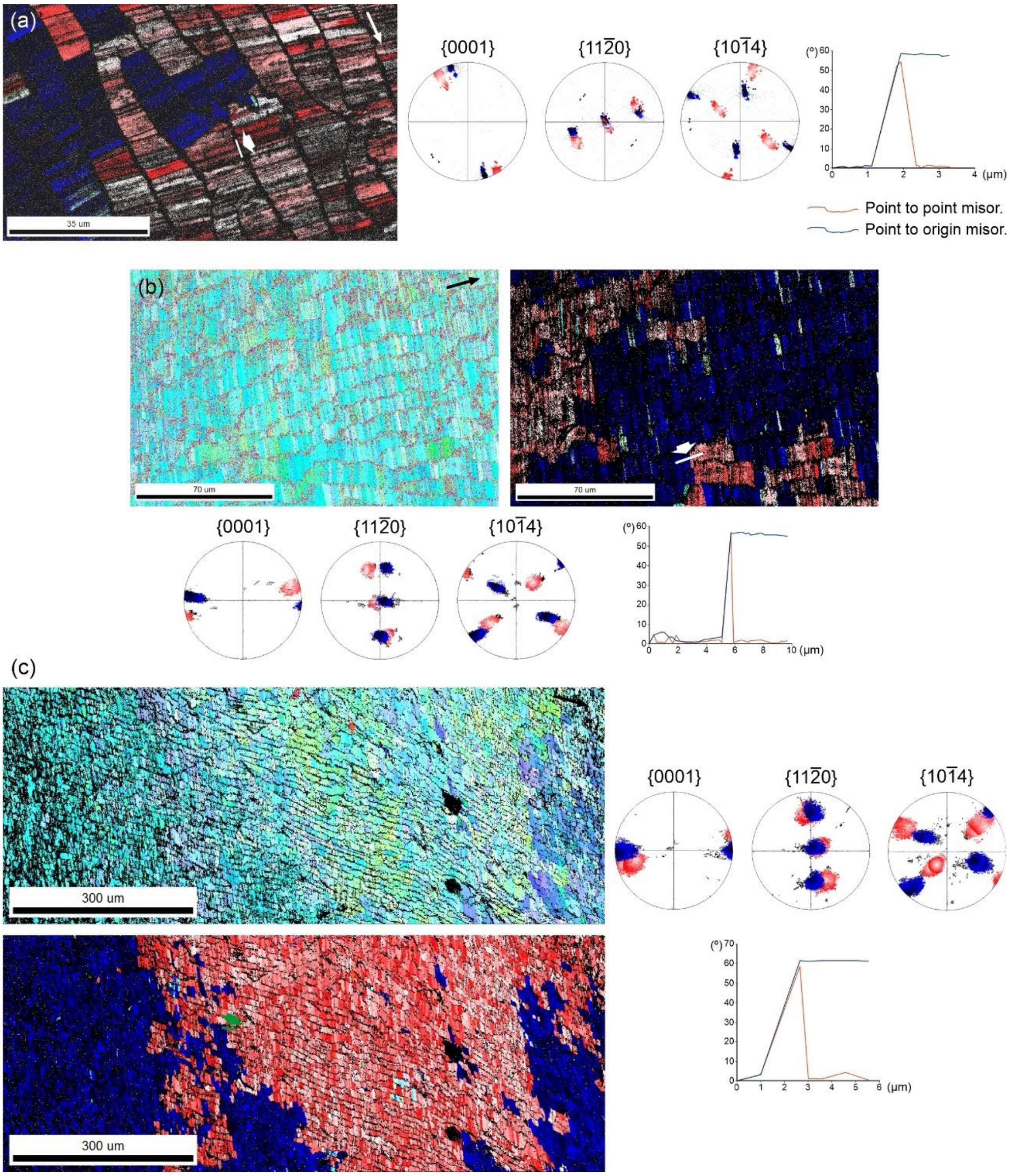

Figure S4. EBSD maps acquired from the unpolished growth surfaces of the foliated layers, revealing a double crystal-like texture. The contribution of different surface areas to the observed maxima is shown both in the red-blue orientation maps and in the associated pole figures. (a) *Cellana toreuma*. See IPF map in Fig. 11b. (b), (c) *Cellana testudinaria*.

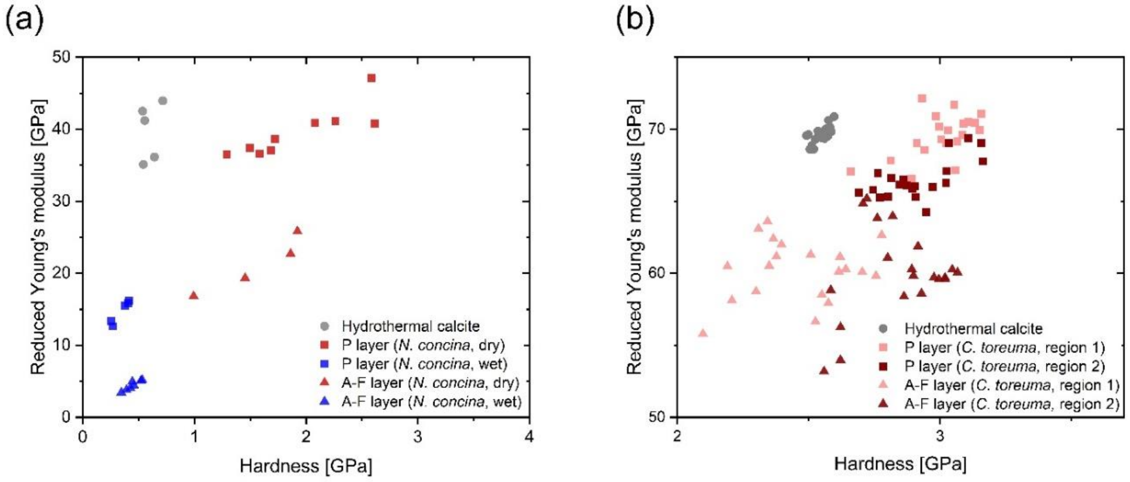

Figure S5. Nanoindentation-derived mechanical properties of nacellid shell layers. (a) Hardness versus reduced Young's modulus obtained from the surface on the outer prismatic (P) and inner acicular–foliated (A–F) of *Nacella concinna* under dry and wet conditions. Red symbols correspond to measurements on dry samples, and blue symbols to hydrated samples, with squares representing the prismatic layer and triangles the A–F layer. Gray circles show values for geological calcite. (b) Hardness versus reduced Young's modulus obtained from cross-section nanoindentation on the prismatic and A–F layers of *C. toreuma* (light and dark red for different regions) and hydrothermal calcite (grey). Squares represent prismatic layer measurements, and triangles represent the A–F layer.

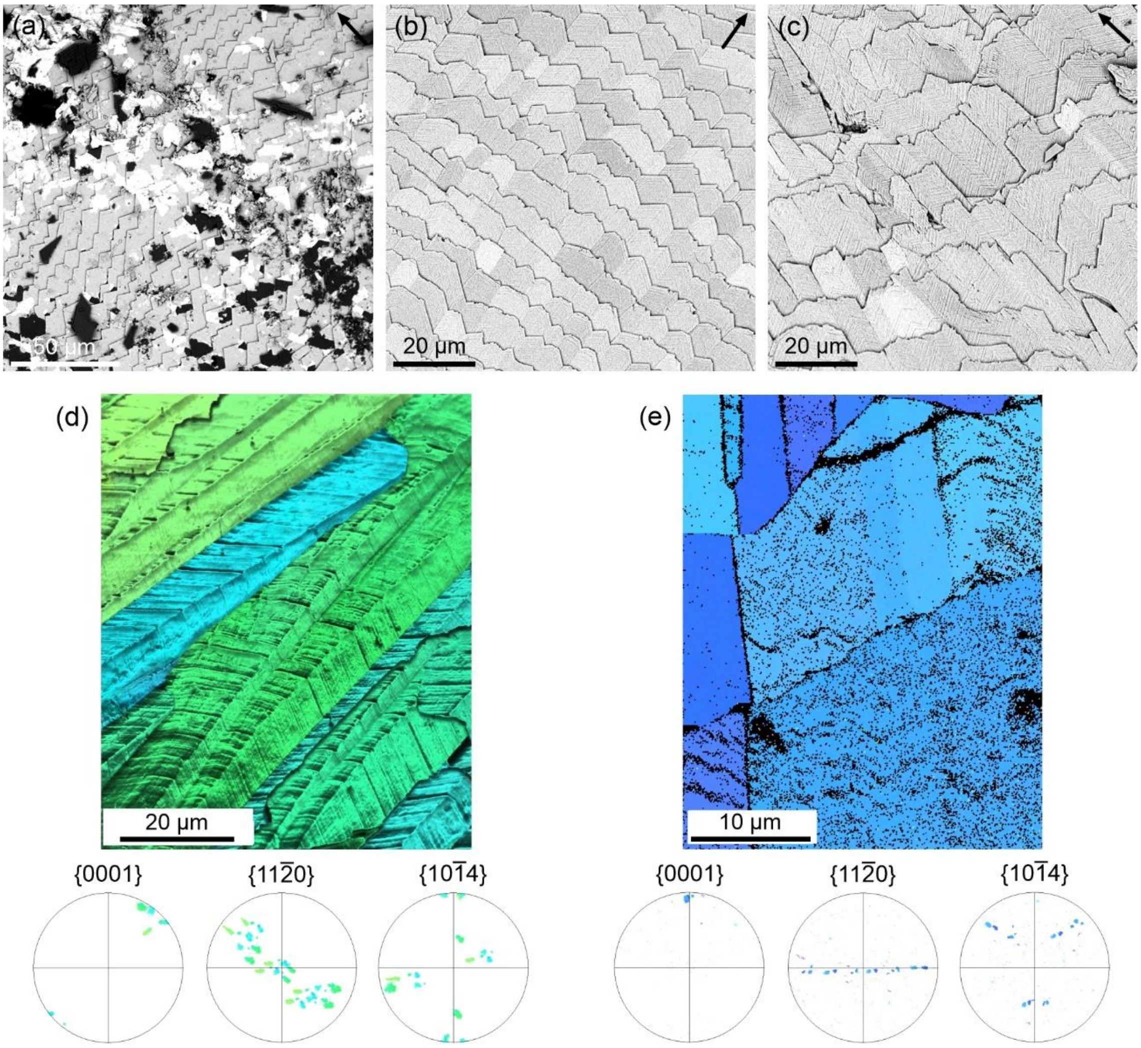

Figure S6. Foliated microstructure of *Eulepetopsis vitrea*. (a)-(c) Surface views of the foliated material, composed of locally co-oriented arrow-pointed laths arranged in extensive folia. (d), (e) Two IPF maps of the surfaces of the folia. The c-axis is parallel to the growth direction of the laths, whereas the remaining crystallographic axes are poorly co-oriented.
